# Coprophagia in early life tunes expression of immune genes after weaning in rabbit ileum

**DOI:** 10.1038/s41598-024-59591-6

**Published:** 2024-04-17

**Authors:** L. Cauquil, M. Beaumont, B. Schmaltz-Panneau, L. Liaubet, Y. Lippi, C. Naylies, L. Bluy, M. Poli, L. Gress, C. Lencina, V. Duranthon, S. Combes

**Affiliations:** 1 GenPhySE, Université de Toulouse, INRAE, ENVT, 31326 Castanet-Tolosan, France; 2 Université Paris-Saclay, UVSQ, INRAE, BREED, 78350 Jouy-en-Josas, France; 3 Ecole Nationale Vétérinaire d’Alfort, BREED, 94700 Maisons-Alfort, France; 4 Toxalim, Université de Toulouse, INRAE, ENVT, INP-Purpan, Toulouse, France

**Keywords:** Immunity, Interferon, Gut, Cytokines, Antimicrobial peptides, Gene expression, Animal behaviour, Animal physiology, Immunology, Physiology, Zoology

## Abstract

Coprophagia by suckling rabbits, i.e. ingestion of feces from their mother, reduces mortality after weaning. We hypothesized that this beneficial effect of coprophagia is immune-mediated at the intestinal level. Therefore, this study investigated immune development after weaning by analyzing the ileal transcriptome at day 35 and 49 in rabbits with differential access to coprophagia in early life. Rabbit pups had access between day 1 and 15 to (i) no feces (NF) or (ii) feces from unrelated does (Foreign Feces, FF) or (iii) feces from unrelated does treated with antibiotics (FFab). 350 genes were differentially expressed between day 35 and day 49 in suckling rabbits with access to coprophagia. These genes coded for antimicrobial peptides, a mucin, cytokines and chemokines, pattern recognition receptors, proteins involved in immunoglobulin A secretion and in interferon signaling pathway. Strikingly, prevention of coprophagia or access to feces from antibiotic-treated does in early life blunted immune development between day 35 et 49 in the ileum of rabbits. Thus, coprophagia might be crucial for the maturation of intestinal immunity in rabbits and could explain why this behavior improves survival.

## Introduction

Unlike other altricial species (cats, rats or mice), mother–pup interactions are extremely limited in rabbit. In both wild and domestic rabbit does^1^, the nursing behaviour lasts less than five minutes and ends with the departure of the doe that jumps out of the nest abruptly and does not return to the nest for 24 h. To overcome this mother-young interaction scarcity, which is probably linked to antipredation behavioural traits, rabbit species have developed specific behaviours and physiological adaptations that lead to enhance survival. For example, the success of the once-daily milk intake is optimised thanks to the secretion in the milk of an olfactory attractive molecule, 2-methyl-2-butenal, a pheromone capable of inducing the typical orocephalic udder-searching behaviour^2^. Coprophagia (i.e. ingestion of feces from the mother) described by Hudson and Distel^1^ and later by Kovacs et al.^3^ is another behavioural adaptation that leads to enhance survival rate^4^. From three days of age, in addition to the nest material, young rabbits ingest maternal fecal pellets deposited by the mother at the time of nursing. Ingestion of maternal feces peaks around day 10 after birth (1–2 feces/day/litter) and continues until day 20, with weaning occurring around 35 days of age in commercial rabbit breeding. Despite the small amount of maternal faecal pellet ingested compared to milk ingestion, coprophagia would provide both nutrients and a mother–offspring bacterial inoculum. Feces in rabbit, correspond to coarse particle (> 0.3 mm in diameter) composed from non digested fibre, endogenous proteins, amino acids and vitamins mainly from microbial origin^5,6^. According to Hirakawa^7^, leporids (including rabbits) ingest feces specially when feed availability is low, suggesting a nutritional role of this behavior. Finally, maternal feces found in the nest could be a vector for vertical transmission of the microbiota from mother to offspring, and might compensate for the short contact time between the mother and offspring in rabbit. It might allow a mother to offspring targeted and early microbial colonization of the digestive tract from the first days of neonatal life. In mammals, the key role of the gut microbiota in health and disease has been extensively studied. First, commensal gut microbes prevent the establishment of opportunistic potentially pathogenic microbes by competing for shared nutrients and ecological niches^8,9^. In addition to this "barrier" function, numerous studies have highlighted the effect of the gut microbiota on the development of the immune system^10^. In particular, the early-life exposure to gut microbiota is critical for the establishment of a normal immune function^11,12^.

We have previously shown that preventing coprophagia in early life delays the implantation dynamics of the microbiota in the cecum (the main fermenting compartment of the rabbit gut) and leads to increased mortality with the strongest effect observed after weaning^4^. Despite a similar ingestion of feces by pups, the beneficial long lasting effect of coprophagia on survival rate was no longer observed when feces came from does treated with antibiotics. Based on these previous results, we hypothesized that the beneficial effect of early life coprophagia on rabbit health involved immune mechanisms in the intestine. The aim of this study was to investigate immune development by analyzing ileal transcriptome at day 35 (age at weaning) and 49 in rabbits with different coprophagia behavior in early life, using the same experimental design as previously described for survival rate and cecal microbiota implantation^4^. Using a rabbit 60 K microarray platform, our study revealed that coprophagia during early life tunes immune maturation in the ileum after weaning which may explain the improved survival rate of pups.

## Results and discussion

In order to study the effect of early life coprophagia on ileal transcriptome in young rabbits (n = 56), the following three groups were studied at 35 (weaning) and 49 days of age. These postnatal days were chosen because we have previously shown, using the same experimental design, that preventing coprophagia in suckling rabbits has long-lasting effects that can be observed after weaning. Preventing coprophagia early in life delays caecal microbiota maturation and reduce survival-rate from 90.7 to 77.2%, with the strongest effects observed between day 35 and day 49^4^. In the NF group (No access to Feces), ingestion of maternal feces in the nest was prevented, while in the two other groups the ingestion of feces by pups was allowed by removing the maternal feces from the nest and replacing them with feces from unrelated does without or with antibiotic supplementation: the FF group (access to foreign doe Feces) and the FFab group (access to unrelated Feces from antibiotics treated doe). After signal intensity normalization and microarray probe annotation improvement (Additional Files 1 and 2) a principal component analysis (PCA) of the 10,554 unique annotated probes was carried out (Additional File 1) and revealed that samples clustered according to age (35 days vs 49 days) but not according to treatments.

**Figure 1:**
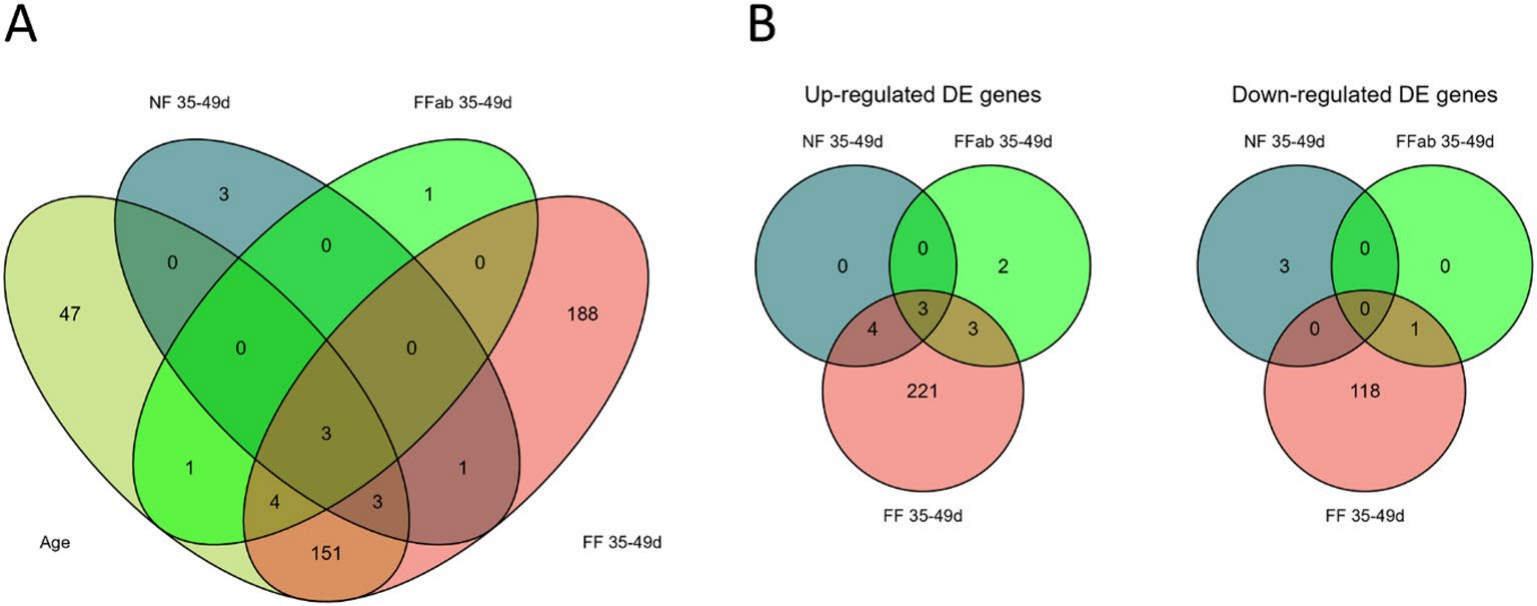
Venn diagram of differentially expressed (DE) genes according (**A**) to the age effect and the 3 contrast analyses and (**B**) for up- and down regulated DE genes between 35 and 49 days of age in NF group, where ingestion of feces was prevented, in the FF and the FFab groups where pups had access in the nest to feces excreted by unrelated does receiving either no antibiotic or treated with tiamulin and tetracycline.

In agreement with the PCA, transcription was affected by age with a total of 209 genes differentially expressed (DE) with thresholds of |log2FC|> 0.5 and P.adj < 0.05 (Table 1, Additional File 3). No DE genes were observed according to treatments when age was not considered. However, contrast analysis revealed that the number of DE genes according to age (35 days vs 49 days) differed between treatments (Fig. 1A). Coprophagia by suckling rabbit leads to the differential expression of 350 genes in the ileum between 35 and 49 days of age (FF group, Additional File 4). A set of 188 genes out of the 350 genes were found DE with age only in the FF group (Contrast FF-35-49d, Fig. 1A). Almost two third of the 350 DE genes of the FF group were upregulated (Contrast FF-35-49d, Table 1 and Fig. 1B). When coprophagia was prevented, the number of DE genes between 35 and 49 days of age was very low (10 DE genes for NF group, Additional File 5) and was equivalent to the number of DE genes of pups that had access to feces from does treated with antibiotics (9 DE genes for the FFab group, Additional File 6). According to age, the log2 fold change (log2FC) values of the ileal transcriptome varied from − 1.96 to 3.06 (Additional File 3) and the age-related DE variability was the highest in the FF group (– 3.37 to 3.96, Table 2, Additional File 4) compare to that in the NF and the FFab group whom log2FC values ranged from − 0.77 to 2.12 and − 0.60 to 1.43 respectively (NF group, Additional file 5, FFab group, Additional File 6).

**Table 1:** Number of genes found up- or down-regulated (|log2FC|> 0.5 and p-adjust value < 0.05) according to age and treatment, and probe gene annotation.

	Genes up-regulated	Genes down-regulated	Total DE genes	Gene annotation	
				HUGO	Ensembl ID
Age	149	60	209	95%	93%
Groups	0	0	0	0	0
Age contrast within groups					
NF between 35 and 49 days	7	3	10	100%	100%
FF between 35 and 49 days	231	119	350	89%	91%
FFab between 35 and 49 days	8	1	9	89%	89%

In the NF group, ingestion of feces was prevented, in the FF and the FFab groups, rabbit pups had access in the nest to feces excreted by unrelated does receiving either no antibiotic or treated with tiamulin and tetracycline.

**Table 2:** Top-ten up- and down-regulated DE genes in the ileum according to age in the FF group where rabbit pups had access in the nest to feces excreted by unrelated does receiving no antibiotics.

Up-regulation			Down-regulation		
Gene symbol	Log2FC	Adj.P.val	Gene symbol	Log2FC	Adj.P.val
REG3G	3.96	3.95E–04	FGF19	− 3.37	3.82E–06
IDO1	3.59	4.17E–02	HMGCS2	− 3.01	1.03E–03
DDX60	3.17	1.57E–04	NPC1L1	− 2.79	4.58E–02
UBD	2.85	5.63E–03	TM4SF4	− 2.38	1.42E–02
GZMH	2.66	1.78E–03	CA1	− 2.20	6.75E–03
USP18	2.40	5.34E–03	EPOR	− 2.02	3.51E–02
HERC5	2.39	7.26E–04	MMP1	− 1.94	2.02E–02
GBP1	2.37	1.16E–03	NR0B2	− 1.83	2.36E–02
LYG2	2.21	1.12E–03	ANXA13	− 1.63	5.45E–03
XAF1	2.18	1.73E–03	NPL	− 1.57	1.21E–03

Altogether, the higher number of DE genes in the FF group between 35 and 49 days of age compared to the NF group suggests that coprophagia early in life modulates ileal maturation. Similarity of gene expression profile (number and level of gene DE) between the NF and the FFab groups suggest that ingestion of feces from antibiotic-treated does might dampen the effects of coprophagia on the gene expression. Does were treated with tetracyclin and tiamulin antibiotics. These antibiotics are used in veterinary medicine and particularly in rabbit farming to treat Epizootic Rabbit Enteropathy^13^. Both were found in the feces of the unrelated treated does (38.2 μg/g feces and 0.97 μg/g feces for tetracycline and tiamulin, respectively^4^). Both have a broad antimicrobial spectrum of activity against gram-negative and gram-positive bacteria. Tetracycline antibiotics bind to the bacterial 30S ribosomal subunit and prevent translation^14^. In the same way, the tiamulin antibiotic targets the 50S subunit of the bacterial ribosome inhibiting protein synthesis^15^. Thus, the presence of these antibiotics in the ingested feces might have altered ileal bacterial community of pups and might have further indirectly impacted their ileal transcriptome. In addition, similar effects of preventing coprophagia and ingesting feces from antibiotic-treated does may indicate that the gene expression effects observed following feces ingestion (FF group) are more likely to be related to the feces bacterial load rather than its nutritional content (feces residual dietary fibers, microbial protein or vitamins^5^). Altogether, the dampening of immune development in the ileum, after weaning, when pups ingested the feces from does treated by antibiotics could thus involve two complementary mechanisms: (i) indirectly through the lack of vertical transmission of bacterial taxa and (ii) directly through antimicrobial action of the residual antibiotics present in feces. To further investigate whether coprophagia in suckling rabbits alters the ileal microbiota composition after weaning, we carried out V3-V4 amplicon sequencing of the 16S RNA genes. Although we observed an effect of age on α and β diversity and taxonomic composition of ileal microbiota, no effect of early life coprophagia was observed on the microbiota after weaning (Additional File 7). We had previously shown, using the same experimental design, that preventing coprophagia delayed cecal microbiota maturation with the strongest effect observed at day 35 and day 49. The difference in results between these two digestive segments could be explained in particular by the greater inter-individual variability in the composition of the microbiota in the ileum compared with the caecum^16^. We hypothesize that coprophagia in suckling rabbit could influence the gut microbiota establishment in both ileum and caecum but that these changes could persist after weaning only in the large intestine due to the overall higher stability of the microbial community in this region. The greater ileal microbial variability compared to cecum microbiota might be explained by the influence of the environmental bacterial metacommunity with the introduction of feed and caecotrophy that take place after weaning (i.e. ingestion of soft feces from the anus) in the rabbit.

**Figure 2:**
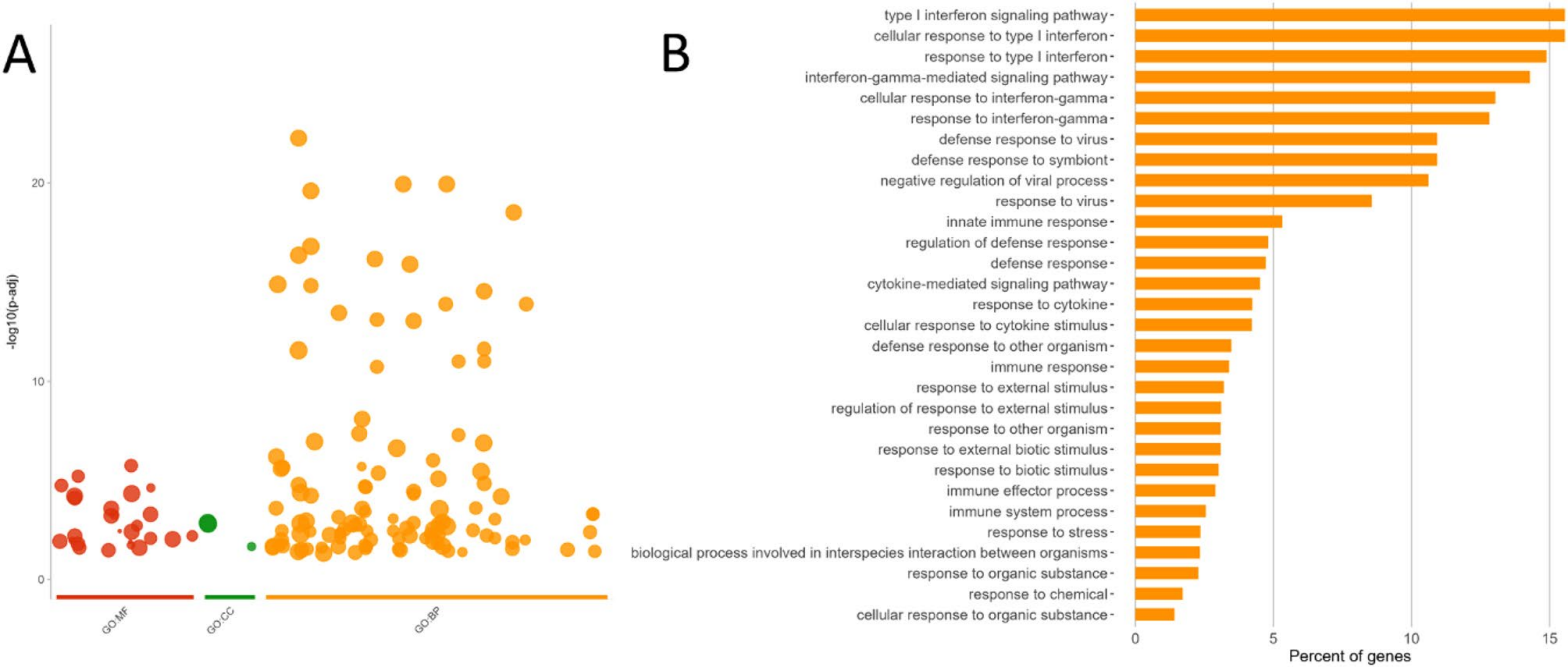
GO analysis of annotated and up-regulated differentially expressed genes in the FF group in the two weeks after weaning where pups had access in the nest to feces excreted by unrelated does not treated with antibiotics. (**A**) Manhattan plot illustrating the significantly changed terms enriched by GO for the Molecular Function (MF), Cellular Component (CC) and Biological Process (BP). The x-axis represents functional terms that are grouped and color-coded by data sources (e.g. Molecular Function from GO is red). The y-axis shows the adjusted enrichment p-values in negative log10 scale. (**B**) Histogram of percentage of enriched genes for the 25 first Biological Process (p adj < 0.025).

In the FF group, early life coprophagia up-regulated genes during the first two weeks after weaning (Table 2, Additional File 4) that code for antimicrobial peptides (REG3G, LYG2), mucin production (MUC13), cytokines and chemokines (CCL4, CCL5, CXCL9, CXCL10, CXCL11, CXCR6, IL15, IL17B, IL18, IFNG, TNFSF10), and some of their binding protein (IL18BP), and receptor (IL18R1), pattern recognition receptors (TLR3, IFIH1), and associated interferon regulatory transcription factor (IRF1, IRF7) and proteins involved in immunoglobulin A secretion (PIGR, TNFSF13, TNFRSF17) or antiviral responses (ISG15, DDX60; HERC5, MX1). To gain functional insight into the FF group up-regulated gene list between 35 and 49 days of age, pathway enrichment analysis was carried out. The 144 significant GO annotations indicated that the enriched biological processes were related to defense response and regulation of defense response, to type I interferon signaling and immune system process (Fig. 2, Additional File 8). The pathway enrichment analysis for the down-regulated gene list 35d-49d FF contrast lead to only 7 significant GO annotations and was not further analyzed.

### Coprophagia in early life enhances type I interferon signaling pathway in the ileum after weaning

**Figure 3:**
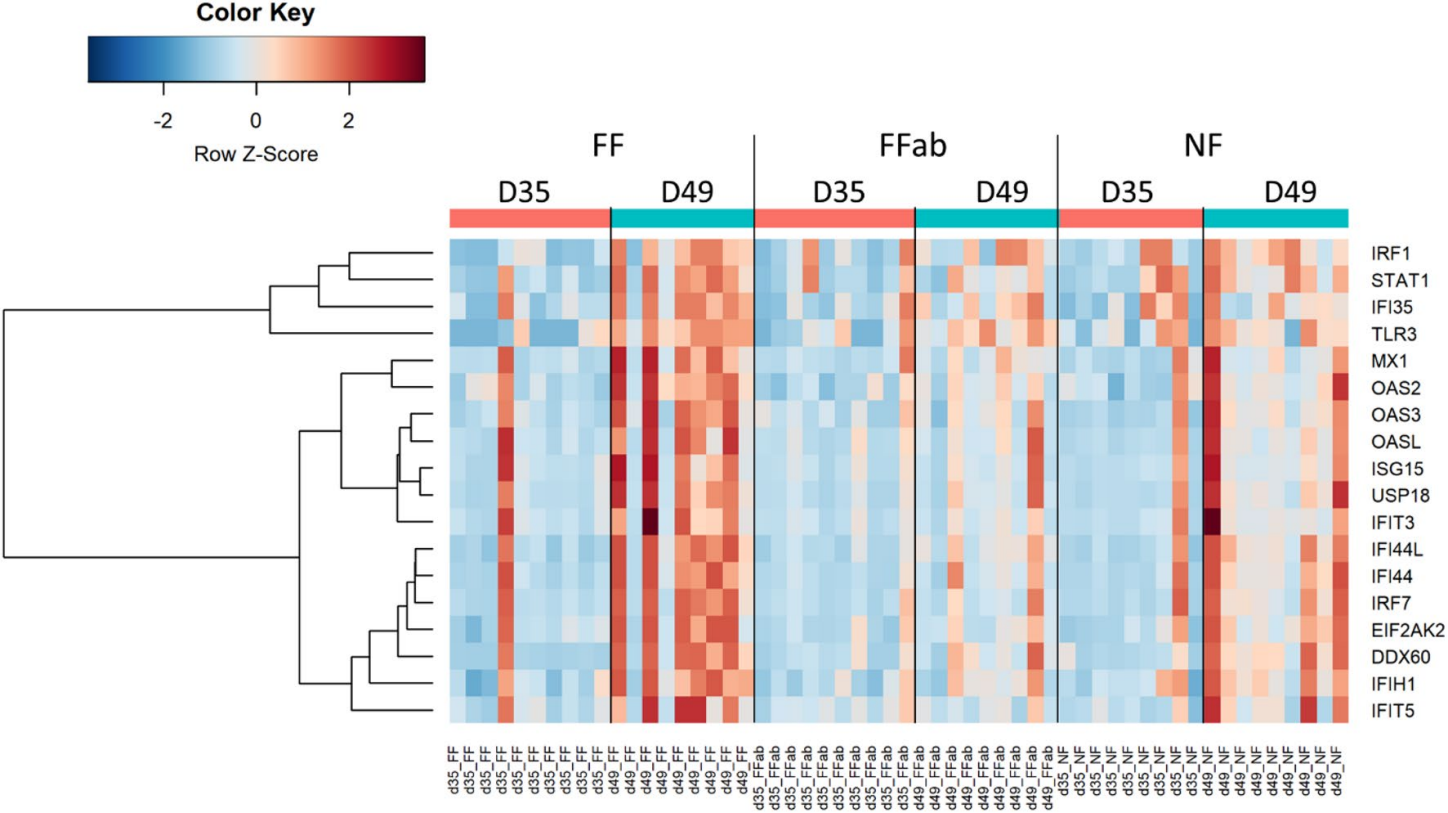
Type I interferon signaling pathway in the ileum of young rabbit after weaning in the NF group, where ingestion of feces was prevented, in the FF and the FFab groups where pups had access in the nest to feces excreted by unrelated does receiving either no antibiotic or treated with tiamulin and tetracycline. Heatmap representing the expression level of genes involved in type I interferon signaling pathway (rows) in individual samples (columns). The colors represent the Z-scores (row-scaled expression) from low (blue) to high values (red). Genes (rows) were clustered by the ward.D method.

Type I interferons (IFN) are central regulators of innate and adaptive immune responses^17^. The production of type I IFN is triggered upon detection of microbial products by pattern recognition receptors (PRR) such as “toll-like receptor 3” (TLR3), “DEAD box and DEAH box helicases” (DEXD-box/DDX60) and “melanoma differentiation-associated gene 5” (MDA5/IFIH1)^17^. TLR3, DDX60 and IFIH1 were up-regulated between D35 and D49 in FF rabbits (+ 1.8, + 9.0 and + 1.7 FC, Fig. 3). Signaling pathways downstream of these PRR activate “interferon regulatory factors” (IRF), and DExH-box helicase 58 (LGP2/DHX58), which acts as a regulator of DDX58/RIG-I and IFIH1^18^. IRF and DHX58 thus regulates the production of type I IFN^17^. IRF1, IRF7 and DHX58 were upregulated between D35 and D49 in FF rabbits (+ 1.7, + 3.8 and + 2.2 FC, respectively). Subsequently, the released type I IFN trigger the binding of “signal transducer and activator of transcription” (STAT1 and STAT2) and IRF9 which together activate the transcription of “interferon-stimulated genes” (ISG)^17^. Type I IFN can also signal through STAT1 homodimers. STAT1 was upregulated between D35 and D49 in rabbits that had ingested feces from doe in early life (+ 2.1 FC). Accordingly, numerous ISG^19^ were also upregulated, including ISG15 (+ 3.9 FC), “ubiquitin specific peptidase 18” (USP18, + 5.3 FC), “myxovirus resistance 1” (MX1,  + 3.3 FC), “IFN-inducible double-stranded RNA-dependent protein kinase” (PKR/EIF2AK2,  + 1.8 FC), “oligoadenylate synthetase” (OAS) OAS2, OAS3, OASL (+ 1.7, 1.7 and 3.1 FC respectively), “interferon induced protein” (IFI) IFI44, IFI35, IFI44L, IFIT3, IFIT5. (+ 3.5, + 2.1, + 2.7, + 3.3 and + 2.1 FC, respectively) and CXC-chemokine ligand 9 (CXCL9)^17^. Overall, our results indicate that type I IFN signaling was strongly activated between D35 and D49 in the ileum of rabbits that ingested feces from doe in early life. We hypothesize that ingestion of adult feces in early life might have resulted in the implantation of bacterial species able to activate type I IFN signaling. Indeed, the gut microbiota plays an essential role in the induction of type I IFN signaling in the gut, as demonstrated by experiments in germ-free or antibiotics treated mice. Depletion of microbiota prior to virus infection blunted the type I IFN response^20^. Various mechanisms underlying the microbial enhancement of type I IFN signaling have been described, including the detection by TLR3 of double-stranded RNA from commensal bacteria of the small intestine^21^ or involving immunomodulatory bacterial metabolites such as desaminotyrosine^22^.

### Coprophagia in early life activates immune responses in the ileum after weaning

**Figure 4:**
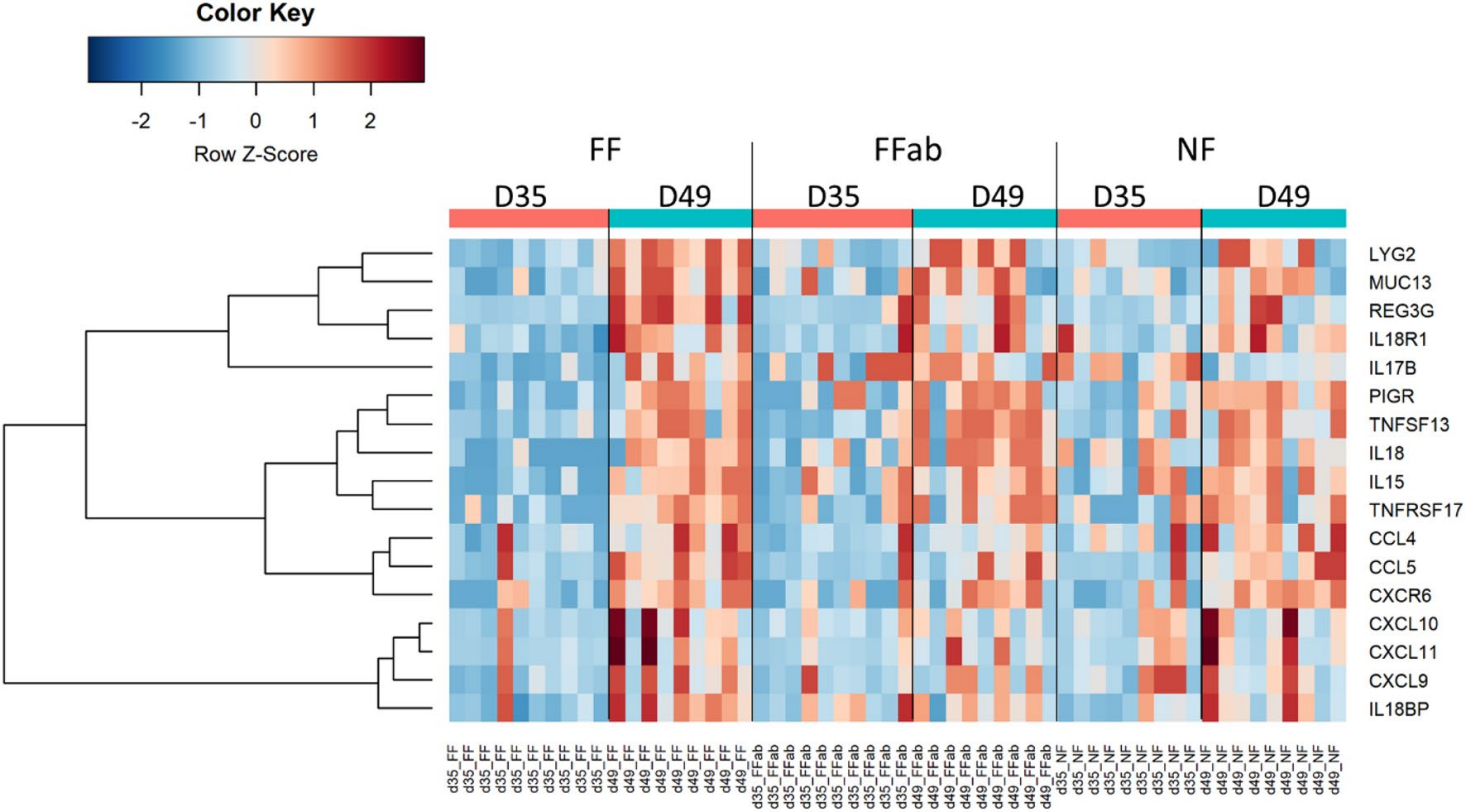
Immune response pathway in the ileum of young rabbits after weaning in the NF group, where ingestion of feces was prevented, in the FF and the FFab groups where pups had access in the nest to feces excreted by unrelated does receiving either no antibiotic or treated with tiamulin and tetracycline. Heatmap representing the expression level of genes involved in immune responses (rows) in individual samples (columns). The colors represent the Z-scores (row-scaled expression) from low (blue) to high values (red). Genes (rows) were clustered by the ward.D method.

The intestinal epithelium plays an important role in the gut barrier function^23^. Epithelial cells limit the quantity of live bacteria at the mucosal surface through the secretion of antimicrobial peptides such as the C-type lectin “regenerating islet-derived protein 3 gamma” (REG3G) or lysozymes^23^. REG3G and “lysozyme G2” (LYG2) were up-regulated between D35 and D49 in FF rabbits (+ 15.6 and + 4.6 FC, respectively, Fig. 4). Epithelial cells also keep bacteria at distance by the expression of transmembrane mucins that form a protective layer called glycocalyx^24^. The transmembrane mucins MUC13 was up-regulated between D35 and D49 in FF rabbits (+ 1.8 FC). Epithelial cells also participate to the secretion of immunoglobulin A (IgA) in the intestinal lumen, which allows the control of the gut microbiota through immune exclusion^25^. Epithelial cells promote naive B cells class-switch recombination to the IgA isotype through the secretion of “proliferation-inducing ligand” (APRIL/TNFSF13) that activates the receptor “B cell maturation antigen” (BCMA/TNFRSF17)^25^. APRIL/TNFSF13 and BCMA/TNFRSF17 were up-regulated between D35 and D49 in FF rabbits (+ 1.7 and + 1.9 FC, respectively). After secretion in the lamina propria by plasmocytes, IgA bind to the polymeric immunoglobulin receptor (PIGR) at the basolateral side of epithelial cells before transcytosis towards the intestinal lumen^25^. PIGR expression was up-regulated between D35 and D49 in FF rabbits (+ 1.9 FC). Ileal IgA levels were further quantified to assess whether upregulation of PIGR could affect its secretion into the lumen. As expected, IgA levels decreased in all groups (Additional File 9), but were not different between groups. This decrease in IgA levels reflects the transition from passive immunity provided by milk ingestion to the development of an endogenous adaptive immune system. According to Paës et al.^26^, a significant increase of caecal IgA concentration after weaning was only observed at postnatal day 57. Thus, in our study, the up-regulation of genes involved in IgA secretion (PIGR, APRIL/TNFSF13, BCMA/TNFRSF17) during the first 15 days after weaning may not be sufficient to translate into IgA levels in the lumen.

Epithelial detection of microbes triggers the secretion of cytokines that recruit and activate innate and adaptive immune cells, which in turn also produce cytokines that, as a whole, regulate mucosal immune responses. Numerous cytokines and related receptors were up-regulated between D35 and D49 in the ileum of FF rabbits: C–C motif ligand 4 (CCL4, + 2 FC), CCL5 (+ 4.3 FC), C-X-C motif chemokine 9 (CXCL9, + 2.6 FC), CXCL10 (+ 3.3 FC), CXCL11 (+ 2.7 FC), C-X-C chemokine receptor type 6 (CXCR6, + 1.7 FC), Interferon γ (IFNG, + 1.5 FC), interleukin 15 (IL15, + 2.2 FC), IL18 (+ 1.6 FC), IL18 receptor 1 (IL18R1, + 1.8 FC), IL18 binding protein (IL18BP, + 1.7 FC) and IL17B (+ 1.6 FC). Altogether, our results indicate a cytokine mediated activation of innate and adaptive immune responses in the ileum of FF rabbits after weaning.

Up-regulation of immune responses in the ileum after weaning has been previously reported in mice and this process is partly dependent on the detection of microbes^27,28^. Accordingly, microbial colonization of germ-free mice induces in the small intestine a strong up-regulation of antimicrobial peptides, genes involved in IgA secretion, cytokines and chemokines^28–32^. Thus, the D35-D49 activation of immune responses in the ileum of FF rabbits might be the result of a high microbial stimuli induced by the early life ingestion of adult microbiota. We previously showed that coprophagia accelerated the establishment of microbiota in the cecum before weaning, allowing bacteria belonging to the Ruminoccocaceae family to became predominant at the expense of those belonging to the Bacteroidaceae family^4^.

### Validation of differentially expressed genes by quantitative reverse transcription-PCR (qRT-PCR) analysis

To validate the microarray results, the expression of a set of 14 genes was analysed using real time qRT-PCR on all the samples (n = 56). These genes were selected according to their expression level (up- or down-regulated, Additional File 10). The similarity between the results obtained with the microarray and qRT-PCR is illustrated in Additional File 9. The 14 genes exhibit a significant spearman correlation between microarray gene and qRT-PCR expression measurement confirming the accuracy of microarray gene (Table 3).

**Table 3:** Correlation between qPCR and microarray results for selected genes (n = 56 samples).

Gene	Spearman’s correlation	P-value
REG3G	0.777	< 0.001
HMGCS2	0.655	< 0.001
DGAT1	0.451	< 0.001
SLC27A4	0.558	< 0.001
LYG2	0.850	< 0.001
MUC12	0.729	< 0.001
KRT20	0.560	< 0.001
FGF19	0.891	< 0.001
NPC1L1	0.837	< 0.001
PIGR	0.634	< 0.001
TNFSF13	0.712	< 0.001
IL18	0.532	< 0.001
IDO1	0.639	< 0.001
NR0B2	0.799	< 0.001

## Conclusions

We have shown that early life coprophagia in rabbits induces transcriptomic changes reflecting immune development in the ileum during the first two weeks after weaning. These changes specifically involve the type I interferon signaling pathway but also include innate immune responses involving altogether transcriptional regulation of antimicrobial peptide, mucin and cytokine secretion and adaptative immune responses through transcriptional regulation of IgA secretion. Lack of differential effect on the ileal transcriptome between 35 and 49 days induced by prevention of early life coprophagia or by ingestion of feces from antibiotic treated does suggest that the effects of coprophagia on ileal maturation after weaning involve vertical transfer of microbes or microbial metabolites. Thus, we propose that the protective effect of early life coprophagia on rabbit survival is mediated by its effects on immune system development in the ileum after weaning. A characterisation of the ileal lamina propria immune cell population would provide additional interesting insights to further understand the effects of early life coprophagia. It would also be interesting to assess whether coprophagia in early life might alter susceptibility to infectious diseases such as epizootic rabbit enteropathy, a common infectious disease affecting rabbits mainly after weaning. Further studies should be conducted to better understand which bacterial taxa or microbial metabolites vertically transmitted from the mother to the pup by coprophagia drive the immune effect of coprophagia. The host-microbiota dialogue is based on intimate interactions involving numerous molecular mechanisms. The recognition of molecular patterns associated with microbes and the action of metabolites resulting from bacterial activity play a role in this context, particularly in the development and maintenance of the integrity of the epithelial barrier and the immune system. Controlling the microbial community building and/or the production of microbial metabolites to optimize this dialogue is a promising avenue of research to promote health around weaning.

## Methods

### Experimental design

All animal housing and handling procedures complied with the guidelines for animal research of the French Ministry of Agriculture and were approved by INRAE Toulouse research center president responsible for the application of ethics in animal experimentation (agreement number C3111316). Rabbit doe and their progeny (PS Hyplus 19 × PS Hyplus 39, commercial hybrids; Hypharm, Roussay, France) were born and reared at the PECTOUL Experimental Unit (INRAE, Castanet-Tolosan, France 10.17180/ftvh-x393). This work was done with the same experimental design used to quantify early life coprophagia and its effects on growth performance, survival rate and cecal microbiota analysis^4^. Briefly, to assess the effect of coprophagia on ileal transcriptome, ileal tissue of 35 and 49 day-old rabbits belonging to the following three groups were used. In the NF group (No access to Feces), ingestion of maternal feces in the nest was prevented, while in the two other groups, FF group (unrelated doe, Foreign Feces) and the FFab group (Foreign Feces from unrelated AntiBiotics treated doe), feces ingestion by the pups was monitored removing the maternal feces from the nest and replacing them with foreign faeces from doe without or with antibiotic supplementation (5, 7, and 9 faeces were provided in each nest from 2 to 13, 14 to 17, and 18 to 20 d, respectively). To prevent or quantify pup fecal pellet ingestion in the nest, initial bedding material (wood shavings and maternal fur) was removed and replaced by a layer of wood shavings and a layer of carded cotton. Suckling was controlled by allowing does access to pups until D20 only once a day for 20 to 30 min in the morning. In all the groups, feces excreted by the mothers in nest boxes were removed immediately after suckling and the cotton layer was removed and replaced with a new one. Foreign fecal pellets were collected from 5 nonlactating, nonpregnant unrelated does from the same husbandry and fed the same experimental diet. After a 15 days adaptation to the diet, fecal pellets were collected for 5 days and frozen. Unrelated does were then treated in drinking water with tetracycline (50 mg/kg body weight) and tiamulin (10 mg/kg body weight). At D35 and D59, ten male or female kits per group were weighed and then euthanized after an injection (20 mg Nesdonal ≅ kg^–1^, Merial, Lyon, France; and 0.3 mL T61 ≅ kg^–1^, Intervet, Beaucouzé, France). Immediately after euthanasia, the ileum was isolated and ileum contents were collected in sterile tubes and stored at − 80 °C until further analysis. The ileal tissue was collected, washed in ice-cold PBS and snap-frozen in liquid nitrogen and stored at − 80 °C. The stomach was isolated and a picture of its contents was taken to confirm the presence or absence of ingested hard fecal pellets.

### Ileal content DNA extraction, 16S rRNA gene sequencing and sequences analysis

Total microbial genomic DNA was extracted from 50 mg of ileal luminal content using the Quick-DNA Fecal/Soil Microbe Miniprep Kit (ZymoResearch, Irvine, California, USA) after mechanical lyses by bead beating, according to manufacturer’s instructions. 16S rRNA gene V3-V4 region was amplified using the primer set 343 F/784 R (343 F: 5′–CTTTCCCTACACGACGCTCTTCCGATCTACGGRAGGCAGCAG –3′ and 784 R: 5′–GGAGTTCAGACGTGTGCTCTTCCGATCTTACCAGGGTATCTAATCCT–3′). Thirty PCR amplification cycles were carried out with an annealing temperature of 65 °C. The PCR products were controlled by gel electrophoresis. The PCR products were sequenced by MiSeq Illumina Sequencing at the Genomic and Transcriptomic Platform (INRAE, Toulouse, France). Amplicon sequences are available online (NCBI accession PRJNA1063899). Sequences processing was performed using the Galaxy-supported pipeline FROGS^33^. Amplicons were filtered according to their size (350–500 nucleotides) and clustered into ASV using Swarm. After chimera removal`, ASV were kept when present in at least 3 samples and representing more than 0.005% and of the total number of sequences^34^. ASV taxonomic affiliation was performed using the reference database silva138 16S with a pintail quality of 100^35^. At the end of the process the mean number of reads per sample in ileal contents was 22 681 (min: 12 200–max: 37 578). The microbiota α and β diversity analyses after rarefaction to even sequencing depth. α diversity indices and relative abundances at the phylum, family and genus taxonomic level were analysed using an ANOVA model.

### RNA extraction

Total RNA was isolated from ileum at 35 and 49 days of age (n = 57 rabbits) as previously described^36^. Ileum samples were homogenized and grounded to a fine powder by rapid agitation for 1 min in a liquid-nitrogen cooled grinder with stainless steel beads. An aliquot of 100 mg of the fine powder was then processed for total RNA isolation and purification using Trizol (Invitrogen, France) and the Nucleospin RNA II kit (Macherey–Nagel, France) according to the manufacturer’s instructions. The method included a DNase digestion step to remove contaminating DNA. The extracted total RNA was eluted in 70 μl of RNase-free water and stored at − 80 °C. RNA quality and concentration were controlled using an AGILENT 2100 bioanalyzer (RNA solutions and RNA 6000 Nano Lab- Chip Kit, Agilent Technologies France, Massy, France).

### Microarray description

The microarray used in this study is a customized DNA Agilent SurePrint G3 Rabbit GE 8 × 60 K (GPL18913 Agilent-042421 Rabbit BDR version 2). That was enriched with a set of 453 specific rabbit genes involved in the immune system process (GO: 0002376)^37^. This microarray contained 62,976 spots. Microarray fingerprints acquisition and microarray data analysis carried out at GeT-TRIX Genopole Toulouse Midi-Pyrénées facility (https://doi.org/10.15454/1.5572370921303193E12). Among them, 1319 were controls spots (Agilent controls). 9029 probes were duplicated twice and 5238 three times. Thus, each array contained 42,089 unique probes targeting 29,026 different genes. After quality control, the signal intensity was found to be above background noise for 33,284 probes, whom 22,599 were Agilent designed probes and 10,685 were custom design probes. The data have been submitted to NCBI GEO (Gene Expression Omnibus; accession number: GSE104838 https://www.ncbi.nlm.nih.gov/geo/query/acc.cgi?acc=GSE104838). After quantile normalization, hierarchical clustering analysis and non-metric multidimensional scaling analysis (nMDS) was carried out to point out outlier sample and one sample was removed from further analysis (Additional File 1). Considering, the continuous improvement of the rabbit genome annotation, probe annotation was improved using an augmented annotation and functional analysis for Agilent gene expression microarray for rabbit, the web tool BetterBunny v3.0 (http://cptweb.cpt.wayne.edu). Finally, the probe annotation was completed with a manual annotation of 36 differentially expressed (DE) probes out of 222 using BLASTn on NCBI database (Additional File 2).

### Microarray statistical analysis

All statistical analyses were performed with R 4.1.0^38^. When several probes targeted one gene, the probe with the median expression level was selected. PCA (principal component analysis) were performed to point out dispersion of the samples. Microarray data were analyzed using the R/Bioconductor package limma (Linear Models for Microarray Data and RNA-seq Data)^39^. A linear model was fitted for all probes using the *lmfit* function for age and treatment, the *makeContrasts* function was used to form a contrast matrix combining age and treatment levels. The fitted model object and contrast matrix were used by the function *contrasts.fit* to compute log_2_-fold-changes and *t*-statistics for all contrasts between age and treatment. Significant differences were determined using Bayes moderated paired t-statistics. Resulting p-values were corrected for multiple testing using the Benjamini–Hochberg False Discovery Rate adjustment. To identify explicit differences between modalities we used cutoff values for a log2fold change (FC) > 0.5 and an adjusted p-value < 0.05.

### Gene ontology enrichment term analysis

Lists of significantly up- and down-regulated genes (absolute log2-fold change greater than 0.5) were analyzed separately for GO enrichment analysis to obtain the top biological functions with g:profiler public web server^40^. The R package gprofiler2 version 0.1.8 which provides an R interface to the g:Profiler tools was used^41^. The tool performs statistical enrichment analysis to find over-representation of information from Gene Ontology terms. gprofiler was used with the following parameters: list of gene name or Human Ensembl Gene ID differentially expressed, Homo sapiens organism, ordered query, no electronic GO annotations, g:SCS multiple testing correction with a threshold at 0.05.

### ELISA measurements of ileal IgA levels

Ileal IgA levels were determined in duplicates by sandwich ELISA in 96-well plates coated with specific polyclonal goat anti-rabbit IgG or IgA antibodies (Bethyl Laboratories, Montgomery, TX, USA) with plate reading at 450 nm as previously described^42^. For IgA relative quantification, samples were pooled to build a reference for the standard curve construction. IgA levels were normalized according to protein concentration and analysed using an ANOVA model.

### Quantitative reverse transcription-PCR (qRT-PCR) analysis and validation of DE genes

cDNA were prepared from 1 µg RNA with Superscript II (ThermoFisher Scientific) following the manufacturer instructions. The primers were designed using Primer 3 software (Additional File 9). Duplicate reactions were performed in a final volume of 5 μL, mixing 2 μL cDNA, 500 nM primers and 2.5 μL SYBR Green PCR Master Mix (Applied Biosystems), using a QuantStudio 12 K Flex system (Life Technologies, Carlsbad, CA, USA). A mix of samples were used to performed a five-point five-fold dilution standard curves for each primer couple to ensure accurate amplification efficiency. Data were analyzed with the 2^–ΔCt^ method with the non-regulated GAPDH gene expression used as a reference. Spearman correlation were calculated between qRT-PCR and microarray expression level.

### Ethics approval and consent to participate

The care, maintenance and experimental use of does rabbit and their progeny (Oryctolagus cuniculus) were performed in accordance with the guidelines for animal research of the French Ministry of Agriculture on the use of agricultural animals in research. This study was approved by INRAE Toulouse research center president responsible for the application of ethics in animal experimentation (number C3111316). This study was performed in accordance with ARRIVE guidelines.

### Supplementary Information

Supplementary Figures.

Supplementary Information 1.

Supplementary Information 2.

Supplementary Information 3.

Supplementary Information 4.

Supplementary Information 5.

Supplementary Information 6.

Supplementary Information 7.

Supplementary Information 8.

Supplementary Information 9.

Supplementary Information 10.

## Data Availability

The files containing information about a microarray experiment are available from the NCBI GEO (accession number: GSE104838). Amplicon sequences are available online (NCBI accession PRJNA1063899). R scripts used to perform statistical analysis can be sourced from GitHub https://forgemia.inra.fr/lcauquil/rabbit-coprophagous-behavior. Additional data generated or analyzed during this study are included within this published article and supplementary files.

## Abbreviations

DE: Differentially expressed
FC: Fold change
qRT-PCR: Quantitative real-time PCR
