# Supplementary material for: Coprophagia in early life tunes expression of immune genes after weaning in rabbit ileum
Source: Sci Rep. 2024 Apr 17;14:8898. doi: 10.1038/s41598-024-59591-6 (PMC11024171; doi:10.1038/s41598-024-59591-6)

**Additional file 1: Additional material for outlier illustrations and PCA of unique annotated expressed probe microarray data**

Based on Hierarchical clustering analysis and Non Metric Distance Scaling (nMDS) plot one sample was considered as outlier and removed from the dataset before further analysis.

**Figure 1** Hierarchical clustering dendogram plot using correlation (1-correlation) as distance matrix and Ward algorithm using all microarray expressed probe with correlation as distance matrix.

**
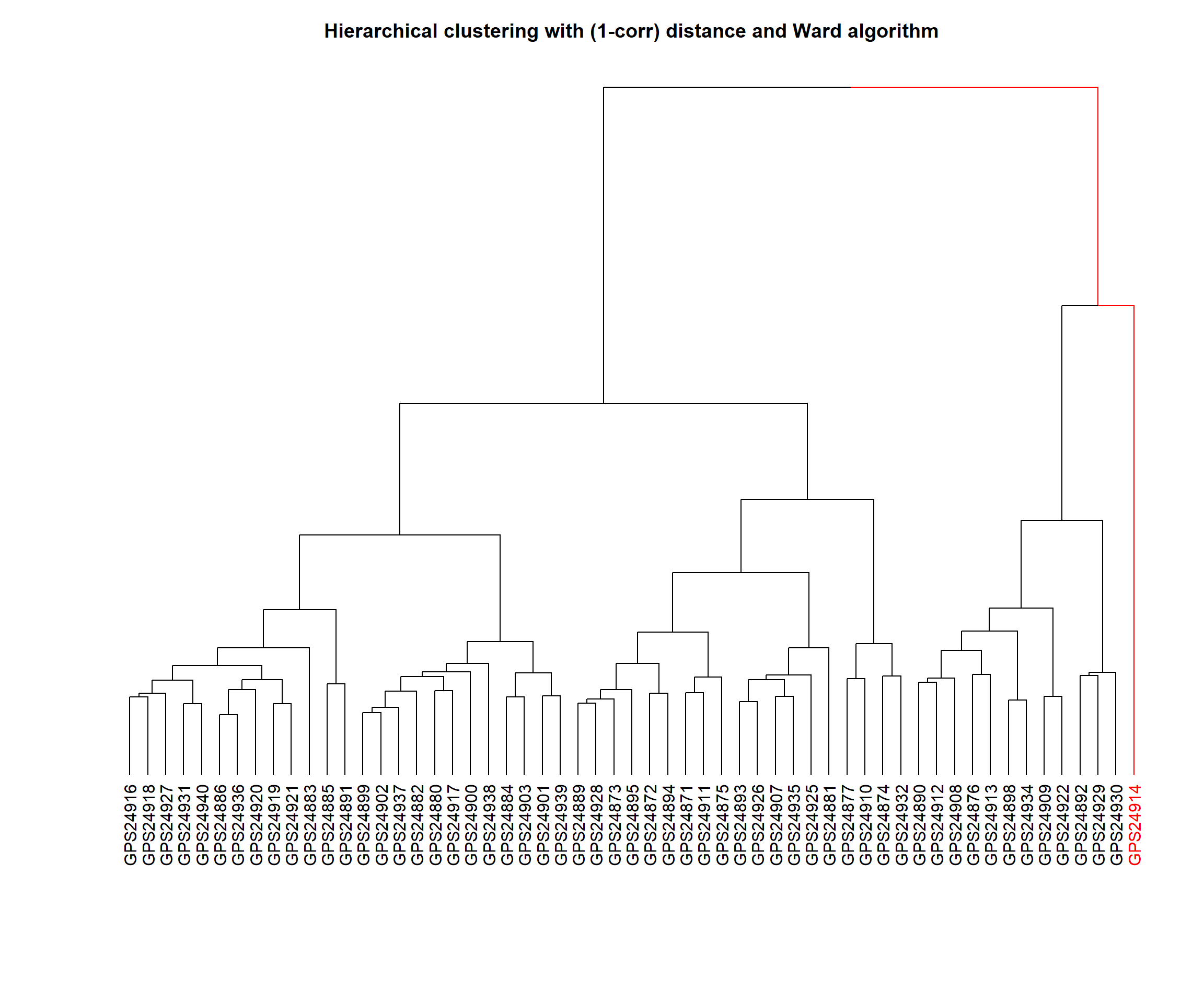
**

**Figure 2** Non Metric Distance Scaling (nMDS) ordination diagram of samples according to age using all microarray expressed probe with 1-correlation as distance matrix.


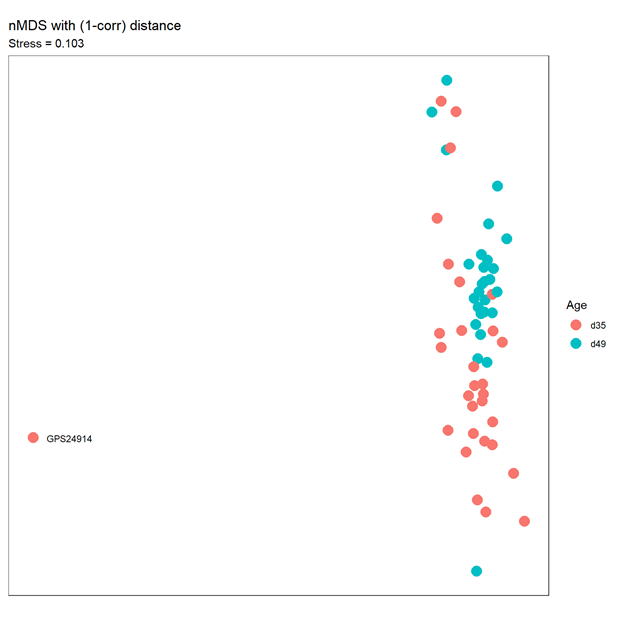


**Figure 3:** Principal component analysis (PCA) of unique annotated expressed probe microarray data according to age (A) or treatment (B). In NF group, ingestion of hard feces was prevented, in the FF and FFab groups pups had access in the nest to feces excreted by foreign females receiving either no antibiotic or medicated with tiamulin and tetracycline


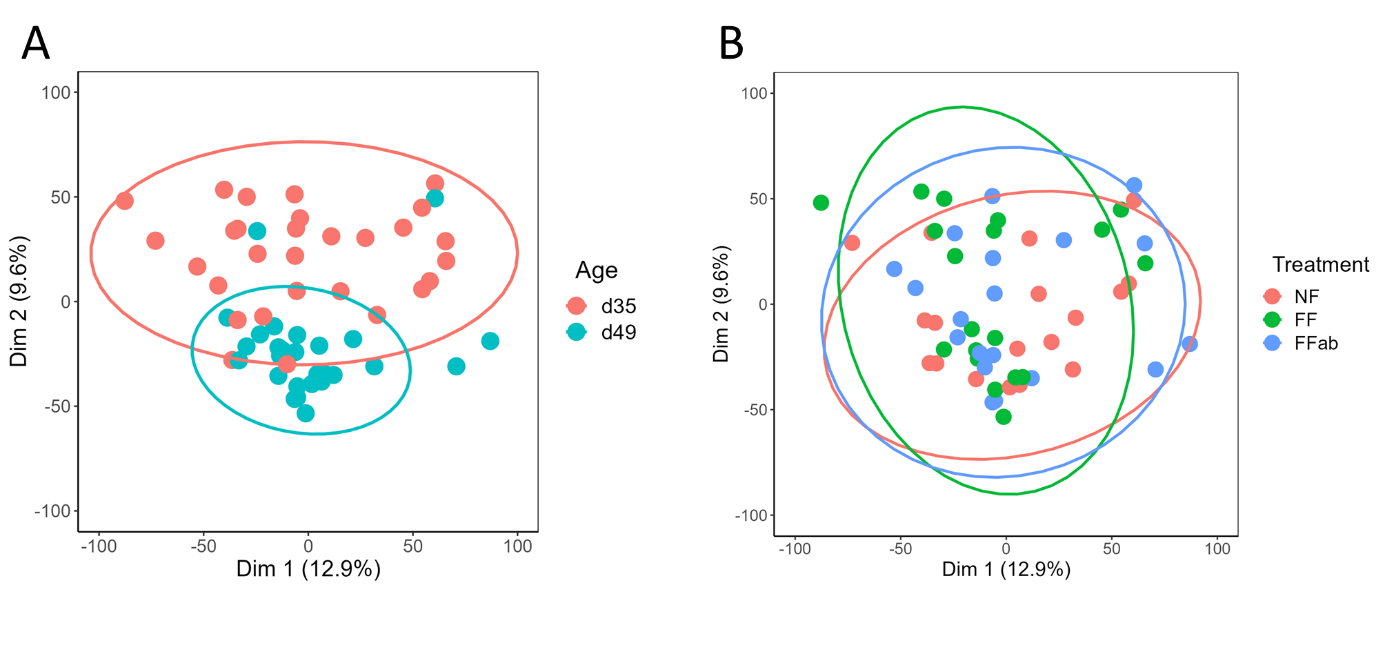

Supplement: Supplementary file 1 — Supplementary Figures. [file 41598_2024_59591_MOESM1_ESM.docx]
