# Supplementary material for: Coprophagia in early life tunes expression of immune genes after weaning in rabbit ileum
Source: Sci Rep. 2024 Apr 17;14:8898. doi: 10.1038/s41598-024-59591-6 (PMC11024171; doi:10.1038/s41598-024-59591-6)

**Additional file 2: Probe annotation pipeline using the Agilent designed probe annotations with BetterBunny augmented annotation and analysis of rabbit genes** (http://cptweb.cpt.wayne.edu)


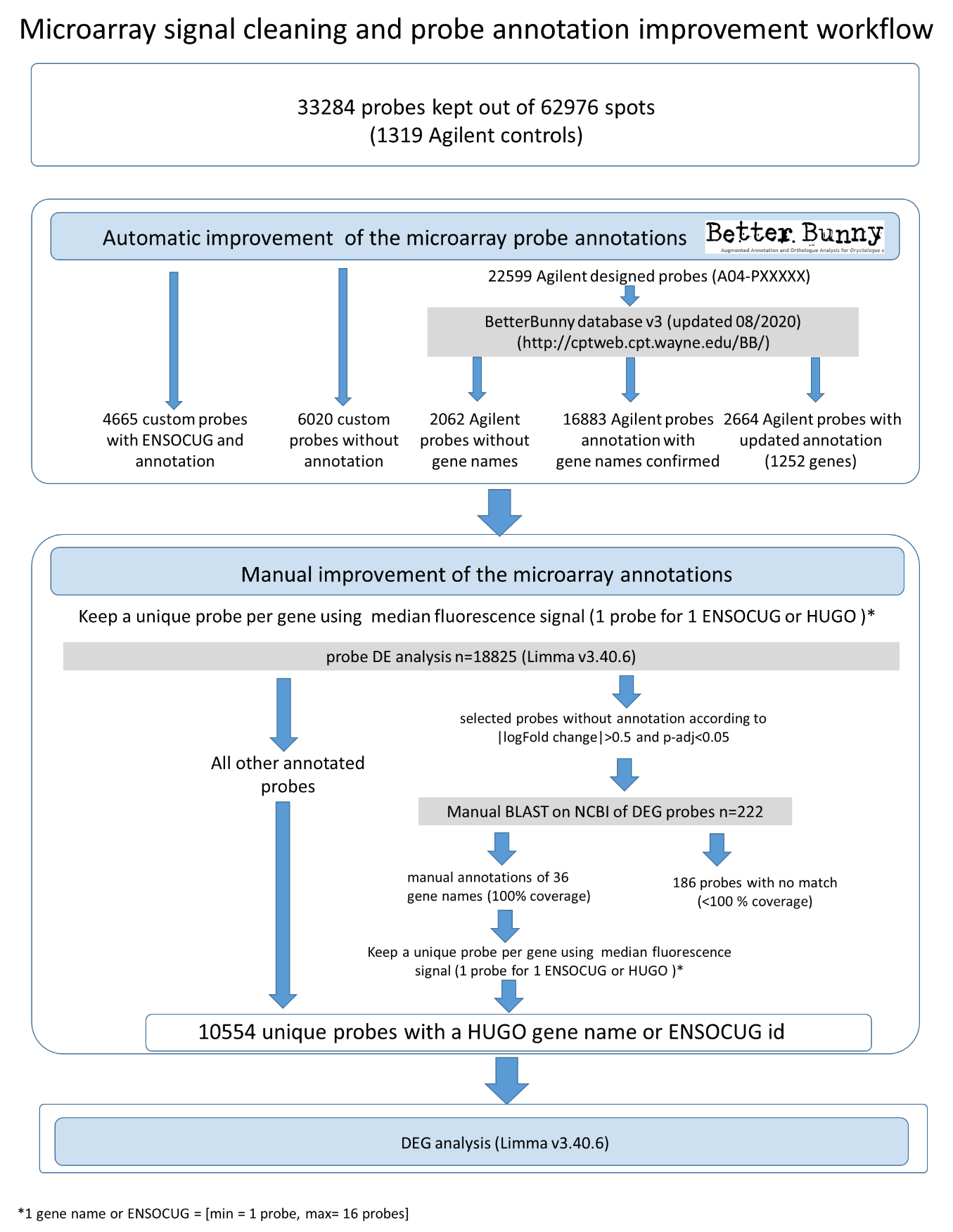

Supplement: Supplementary file 2 — Supplementary Information 1. [file 41598_2024_59591_MOESM2_ESM.docx]
