# Supplementary material for: Coprophagia in early life tunes expression of immune genes after weaning in rabbit ileum
Source: Sci Rep. 2024 Apr 17;14:8898. doi: 10.1038/s41598-024-59591-6 (PMC11024171; doi:10.1038/s41598-024-59591-6)
Supplement: Supplementary file 7 — Supplementary Information 6. [file 41598_2024_59591_MOESM7_ESM.docx]

**Additional file 7: Microbiota alpha, beta diversity and taxonomic composition in ileal content of rabbit at 35 and 49 days of age** in NF group, where ingestion of hard feces was prevented, in the FF and FFab groups where pups had access in the nest to feces excreted by foreign females receiving either no antibiotic or medicated with tiamulin and tetracycline.

**A. Alpha diversity**


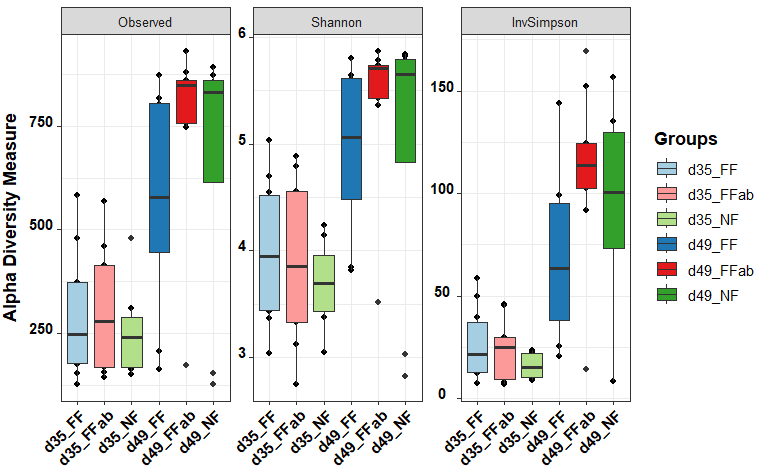


ANOVA table

| **Indices** | **Treatment** | **Age** | **Treatment:age** |
| --- | --- | --- | --- |
| Observed | 0.3631 | <0.001 | 0.4126 |
| Shannon | 0.5223 | <0.001 | 0.5831 |
| InvSimpson | 0.2281 | <0.001 | 0.1478 |

**B. Beta diversity: PCoA plot**


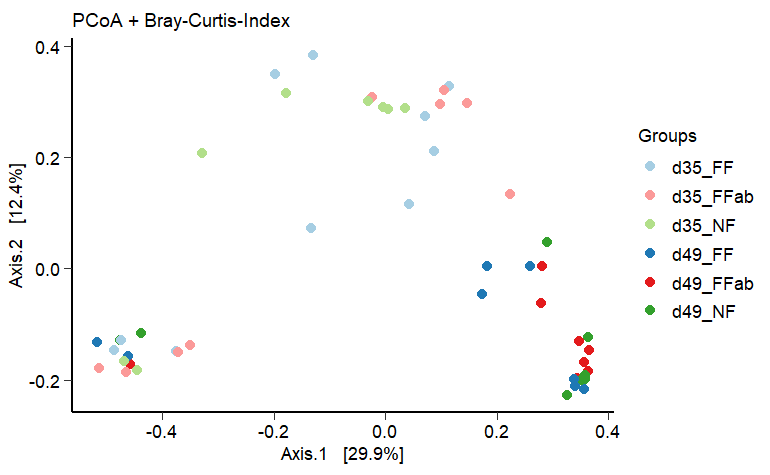


**C. Taxonomic composition**

At phylum level


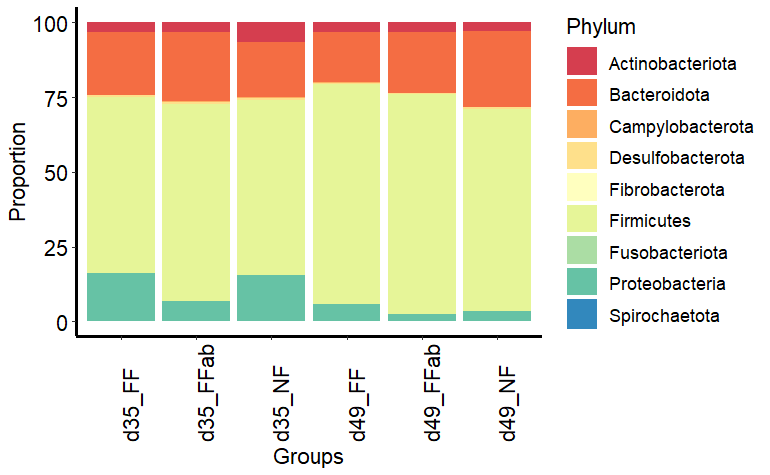


**ANOVA table**

| **Phylum** | **Pvalue treatment** | **Pvalue  age** | **Pvalue treatment:age** |
| --- | --- | --- | --- |
| Actinobacteriota | 0.8379 | 0.7791 | 0.6017 |
| Bacteroidota | 0.5123 | 0.8893 | 0.0716 |
| Campylobacterota | 0.7542 | 0.2933 | 0.8315 |
| Desulfobacterota | 0.7738 | 0.178 | 0.6732 |
| Fibrobacterota | 0.5528 | 0.1289 | 0.5528 |
| Firmicutes | 0.3122 | 0.0043 | 0.6546 |
| Fusobacteriota | 0.9924 | 0.2275 | 0.913 |
| Proteobacteria | 0.1608 | 0.0084 | 0.8032 |
| Spirochaetota | 0.4578 | 0.2646 | 0.2624 |
| Firmicutes to_bacteroides ratio | 0.5332 | 0.4851 | 0.0506 |

At family level (top 20 most abundant families)

**
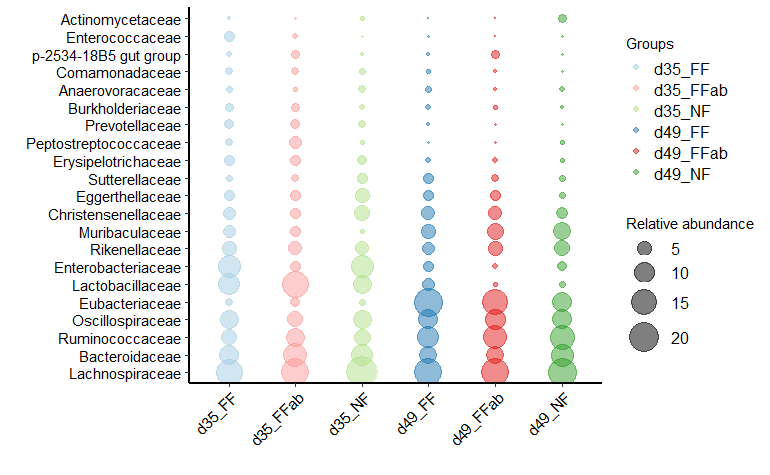
**

**ANOVA table**

| **Family** | **Pvalue treatment** | **Pvalue age** | **Pvalue treatment:age** |
| --- | --- | --- | --- |
| [Eubacterium] coprostanoligenes group | 0.866 | 0.2246 | 0.7639 |
| Actinomycetaceae | 0.2368 | 0.5022 | 0.4733 |
| Anaerovoracaceae | 0.1462 | 0.1568 | 0.8065 |
| Atopobiaceae | 0.9383 | 0.6911 | 0.1884 |
| Bacteroidaceae | 0.478 | 0.3355 | 0.5197 |
| Barnesiellaceae | 0.46 | 0.7701 | 0.6327 |
| Burkholderiaceae | 0.6104 | 0.0658 | 0.8026 |
| Butyricicoccaceae | 0.0552 | 0.9055 | 0.736 |
| Christensenellaceae | 0.6621 | 0.947 | 0.1592 |
| Clostridiaceae | 0.4003 | 0.1507 | 0.4341 |
| Comamonadaceae | 0.657 | 0.0046 | 0.8723 |
| Desulfovibrionaceae | 0.8753 | 0.1481 | 0.9951 |
| Eggerthellaceae | 0.9453 | 0.2584 | 0.3645 |
| Enterobacteriaceae | 0.0885 | 0.0036 | 0.3051 |
| Enterococcaceae | 0.6011 | 0.2845 | 0.5856 |
| Erysipelotrichaceae | 0.9771 | 0.0879 | 0.9931 |
| Eubacteriaceae | 0.336 | 0 | 0.3948 |
| Lachnospiraceae | 0.251 | 0.6258 | 0.7159 |
| Lactobacillaceae | 0.6724 | 0.0091 | 0.5145 |
| Monoglobaceae | 0.6285 | 0.0043 | 0.0855 |
| Muribaculaceae | 0.8399 | 0.0004 | 0.163 |
| Oscillospiraceae | 0.7682 | 0.0777 | 0.3213 |
| p-2534-18B5 gut group | 0.1921 | 0.7844 | 0.974 |
| Peptostreptococcaceae | 0.4605 | 0.1253 | 0.3639 |
| Prevotellaceae | 0.8602 | 0.0338 | 0.8773 |
| Rikenellaceae | 0.843 | 0.7468 | 0.5329 |
| Ruminococcaceae | 0.4192 | 0.0002 | 0.7117 |
| Sutterellaceae | 0.706 | 0.98 | 0.2161 |
| Tannerellaceae | 0.5543 | 0.0063 | 0.9413 |
| UCG-010 | 0.627 | 0 | 0.2343 |

At genus level (top 20 most abundant genera)

**
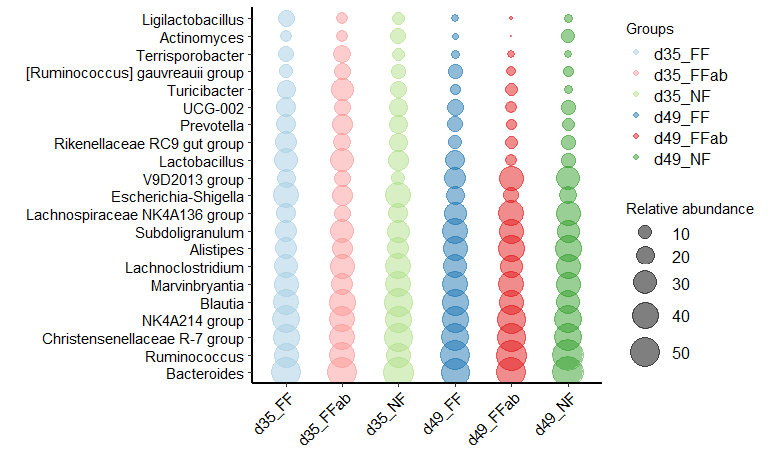
**

**ANOVA table**

| **Genus** | Pvalue treatment | Pvalue  age | Pvalue treatment:age |
| --- | --- | --- | --- |
| [Eubacterium] siraeum group | 0.7884 | 0.3025 | 0.8637 |
| [Ruminococcus] gauvreauii group | 0.9391 | 0.2293 | 0.4434 |
| Actinomyces | 0.1309 | 0.1901 | 0.9023 |
| Alistipes | 0.8834 | 0.0054 | 0.6552 |
| Bacteroides | 0.4675 | 0.9488 | 0.8053 |
| Blautia | 0.9954 | 0.1029 | 0.274 |
| Burkholderia-Caballeronia-Paraburkholderia | 0.4799 | 0.7906 | 0.8551 |
| CAG-352 | 0.6239 | 0 | 0.7153 |
| Christensenellaceae R-7 group | 0.7642 | 0.276 | 0.1481 |
| Clostridium sensu stricto 1 | 0.7777 | 0.0438 | 0.3132 |
| Coprococcus | 0.5174 | 0.5029 | 0.5601 |
| Desulfovibrio | 0.4319 | 0.442 | 0.5788 |
| dgA-11 gut group | 0.9751 | 0.1237 | 0.3778 |
| Enterococcus | 0.5362 | 0.0859 | 0.5543 |
| Escherichia-Shigella | 0.1826 | 0.0029 | 0.7577 |
| Family XIII AD3011 group | 0.5457 | 0.0002 | 0.5181 |
| Fusicatenibacter | 0.9645 | 0.6831 | 0.3388 |
| GCA-900066575 | 0.786 | 0.0044 | 0.8363 |
| Lachnoclostridium | 0.498 | 0.3734 | 0.728 |
| Lachnospiraceae NK4A136 group | 0.8878 | 0.0094 | 0.4449 |
| Lachnospiraceae UCG-006 | 0.2868 | 0.2235 | 0.0979 |
| Lactobacillus | 0.4163 | 0.0108 | 0.536 |
| Ligilactobacillus | 0.3961 | 0.015 | 0.5092 |
| Limosilactobacillus | 0.8852 | 0.0023 | 0.9679 |
| Marvinbryantia | 0.6052 | 0.1317 | 0.1329 |
| Monoglobus | 0.9145 | 0.0103 | 0.2281 |
| NK4A214 group | 0.8752 | 0.3639 | 0.2433 |
| Parabacteroides | 0.5092 | 0.0045 | 0.9766 |
| Prevotella | 0.787 | 0.0075 | 0.6979 |
| Rikenellaceae RC9 gut group | 0.7005 | 0.0421 | 0.7225 |
| Romboutsia | 0.9841 | 0.0802 | 0.789 |
| Roseburia | 0.4476 | 0.0339 | 0.5307 |
| Ruminococcus | 0.7148 | 0 | 0.927 |
| Subdoligranulum | 0.5454 | 0.2538 | 0.3833 |
| Terrisporobacter | 0.8009 | 0.0201 | 0.7328 |
| Turicibacter | 0.2746 | 0.0029 | 0.7291 |
| Tyzzerella | 0.755 | 0.1878 | 0.7074 |
| UCG-002 | 0.6423 | 0.1785 | 0.9685 |
| UCG-005 | 0.9345 | 0.4686 | 0.7956 |
| V9D2013 group | 0.8492 | 0.0013 | 0.3572 |
| Variovorax | 0.972 | 0.2259 | 0.8767 |
