# Supplementary material for: Coprophagia in early life tunes expression of immune genes after weaning in rabbit ileum
Source: Sci Rep. 2024 Apr 17;14:8898. doi: 10.1038/s41598-024-59591-6 (PMC11024171; doi:10.1038/s41598-024-59591-6)
Supplement: Supplementary file 9 — Supplementary Information 8. [file 41598_2024_59591_MOESM9_ESM.docx]

**Additional file 8: IgA levels in ileal content of rabbit after weaning** in NF group, where ingestion of hard faeces was prevented, in the FF and FFab groups where pups had access in the nest to faeces excreted by foreign females receiving either no antibiotic or medicated with tiamulin and tetracycline.


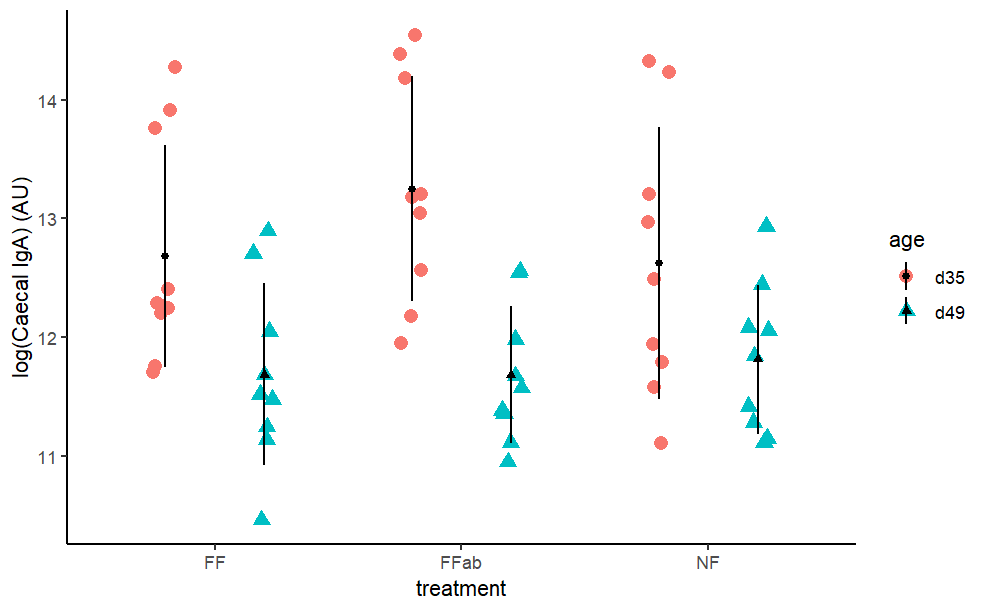


ANOVA table

| **term** | **p.value** |
| --- | --- |
| treatment | 0.561 |
| age | <0.001 |
| treatment:age | 0.393 |
