# Supplementary material for: Coprophagia in early life tunes expression of immune genes after weaning in rabbit ileum
Source: Sci Rep. 2024 Apr 17;14:8898. doi: 10.1038/s41598-024-59591-6 (PMC11024171; doi:10.1038/s41598-024-59591-6)
Supplement: Supplementary file 10 — Supplementary Information 9. [file 41598_2024_59591_MOESM10_ESM.docx]

**Additional file 9: Primer sequences for qRT-PCR validation and graphical representation of selected gene expression using qRT-PCR or microarray and their correlation**

Table 1: Primer sequences for qRT-PCR validation

| Gene symbol | Gene name | Sequence (5’ – 3’)  F: Forward ; R: Reverse |
| --- | --- | --- |
| REG3G | Regenerating family member 3 gamma | F: ATGGACATGGATGGGAGTGG  R: GGGTAACTGCGCATCACAAT |
| HMGCS2 | 3-Hydroxy-3-Methylglutaryl-CoA Synthase 2 | F: AAATCTGGCCTCCGAGTACC  R: GGACCATCTTGCAAAAGGGG |
| DGAT1 | Diacylglycerol O-Acyltransferase 1 | F: GCATCCTGAACTGGTGTGTG  R: TCAGGAACAAAGACACCACC |
| SLC27A4 | Solute Carrier Family 27 Member 4 | F: CTGCTCTACTTGGGGTCTGG  R: GCACTTTGGCCTTCACCTTC |
| LYG2 | Lysozyme G2 | F: TGGGGCGCTCTGTAACATAA  R: GGGTCTACGCAATGCCTCTG |
| MUC12 | Mucin 12, Cell Surface Associated | F: ACGTGTCTCAAGAGTGGCAG  R: GGCCGGAAGTAGCTGTAGAC |
| KRT20 | Keratin 20 | F: GCTGCAGAACCAGATTAAGGATGC  R: GCTAGGCGTATCGCCCTCTC |
| FGF19 | Fibroblast Growth Factor 19 | F: CGCACCGTGGCCTTCAAG  R: CTTGGCACTGCTCAGAGAC |
| NPC1L1 | NPC1 Like Intracellular Cholesterol Transporter 1 | F: CAGCAAATTCCAGGTCACGT  R: CAGCGTGATCTTGGAGTCCA |
| PIGR | Polymeric Immunoglobulin Receptor | F: GGAGCCATTGACAACCCAAG  R: CTTGGGCTCCTCAATCTCCA |
| TNFSF13 | TNF Superfamily Member 13 | F: CACTTCTTCACCTTGTTCCCA  R: CCGGGGAATTACGACACTCA |
| IL18 | Interleukin 18 | F: TGTAAGCCTCTCTGTGAAGTGT  R: TCTTATCTTTCTGTCCTGCGAGA |
| IDO1 | Indoleamine 2,3-Dioxygenase 1 | F: AATGCAGCCCCACTTTCTACTTA  R: TAGCGTCTGTGCTCCAAGTCG |
| NR0B2 | Nuclear Receptor Subfamily 0 Group B Member 2 | F: AATTCGCCGACTCTGACTCT  R: TTTTCGAGGACTGGGACTCC |

Figure 1: Graphical representations of the expression measurement of the 14 selected genes using qRT-PCR (left panel) or microarray (right panel) and their correlation

| 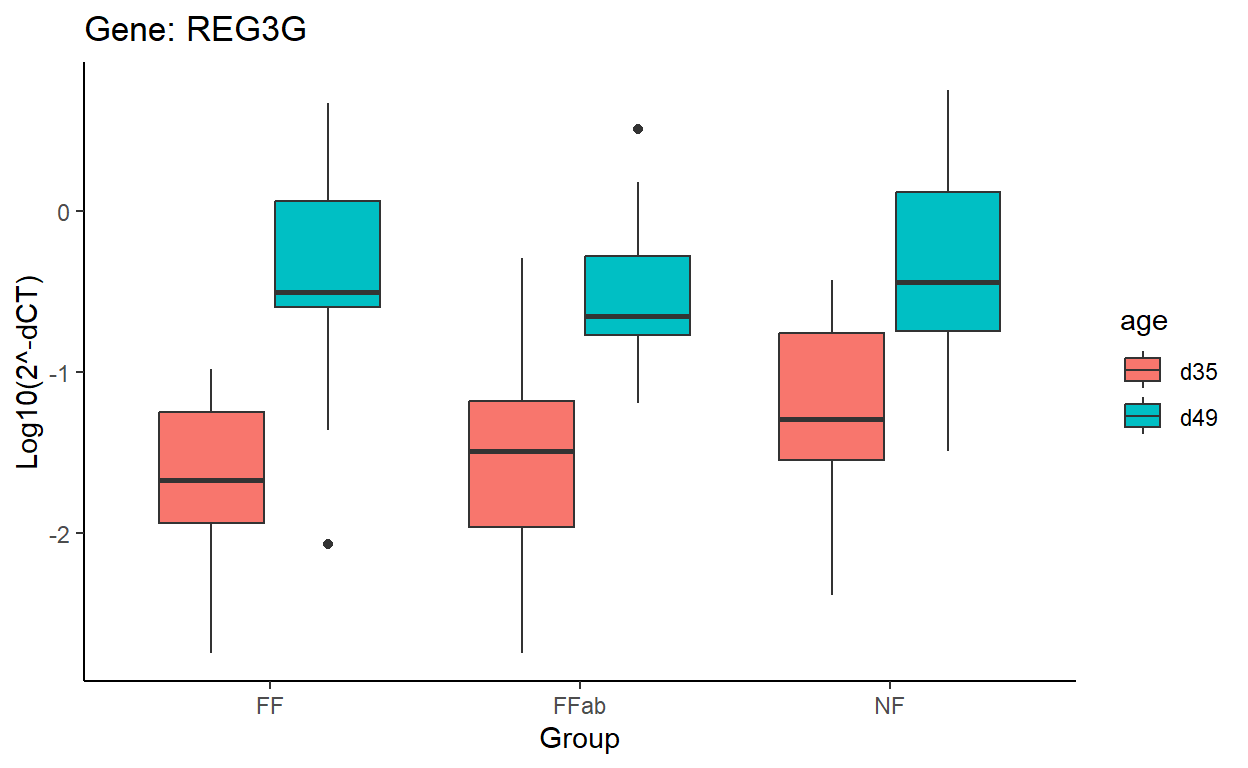 | 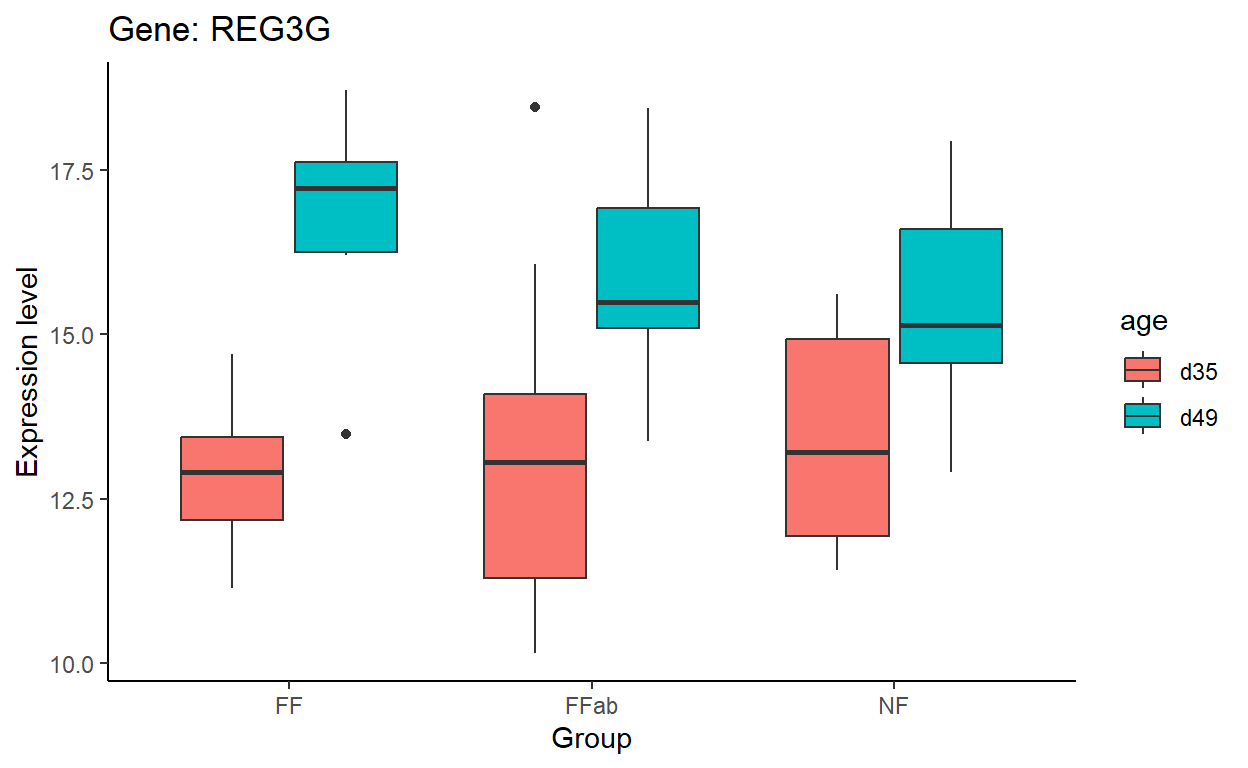 |
| --- | --- |
| 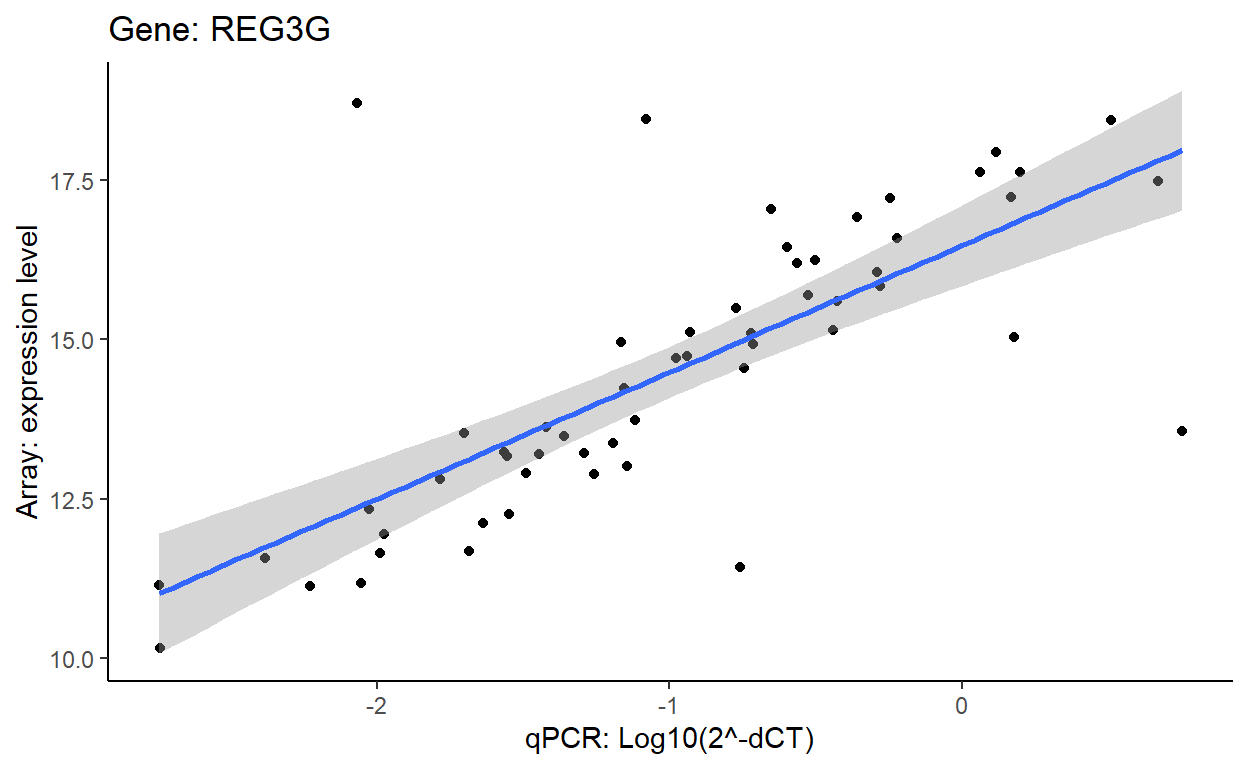 | Spearman's rank correlation rho  p-value = 1.766e-12  rho = 0.7777987 |

| 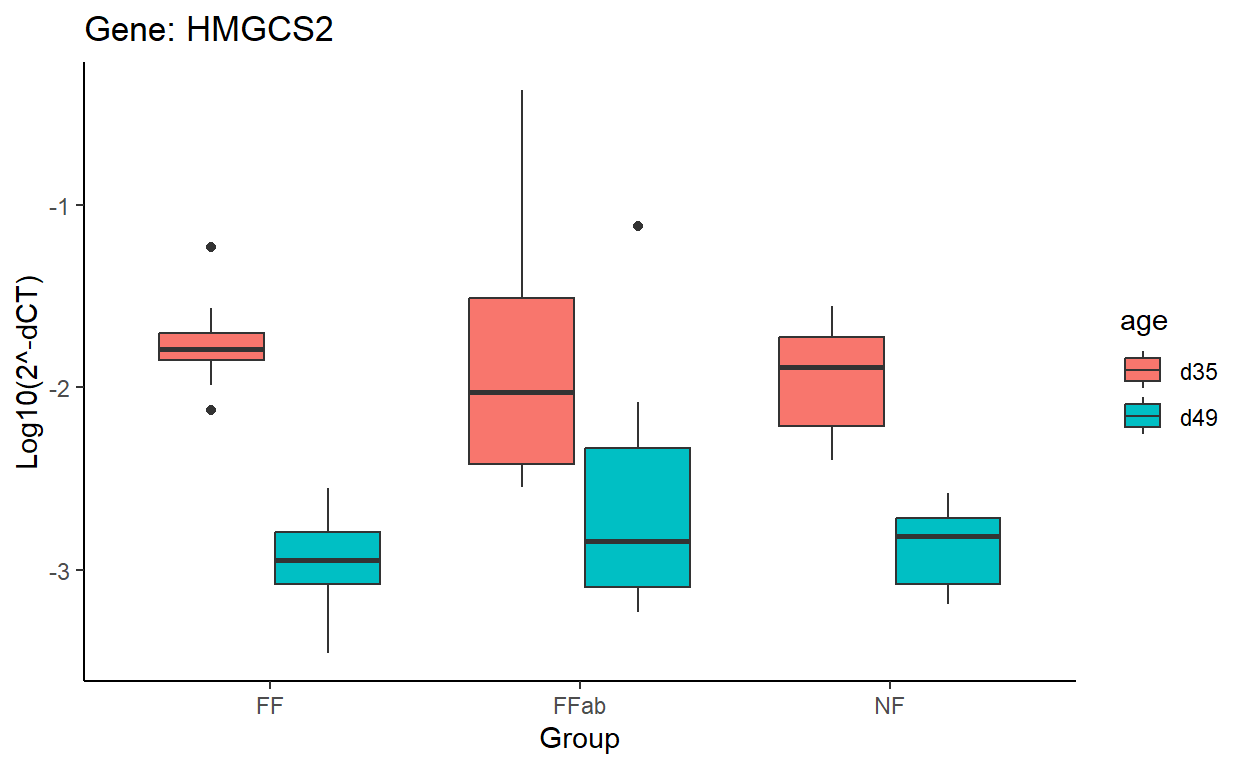 | 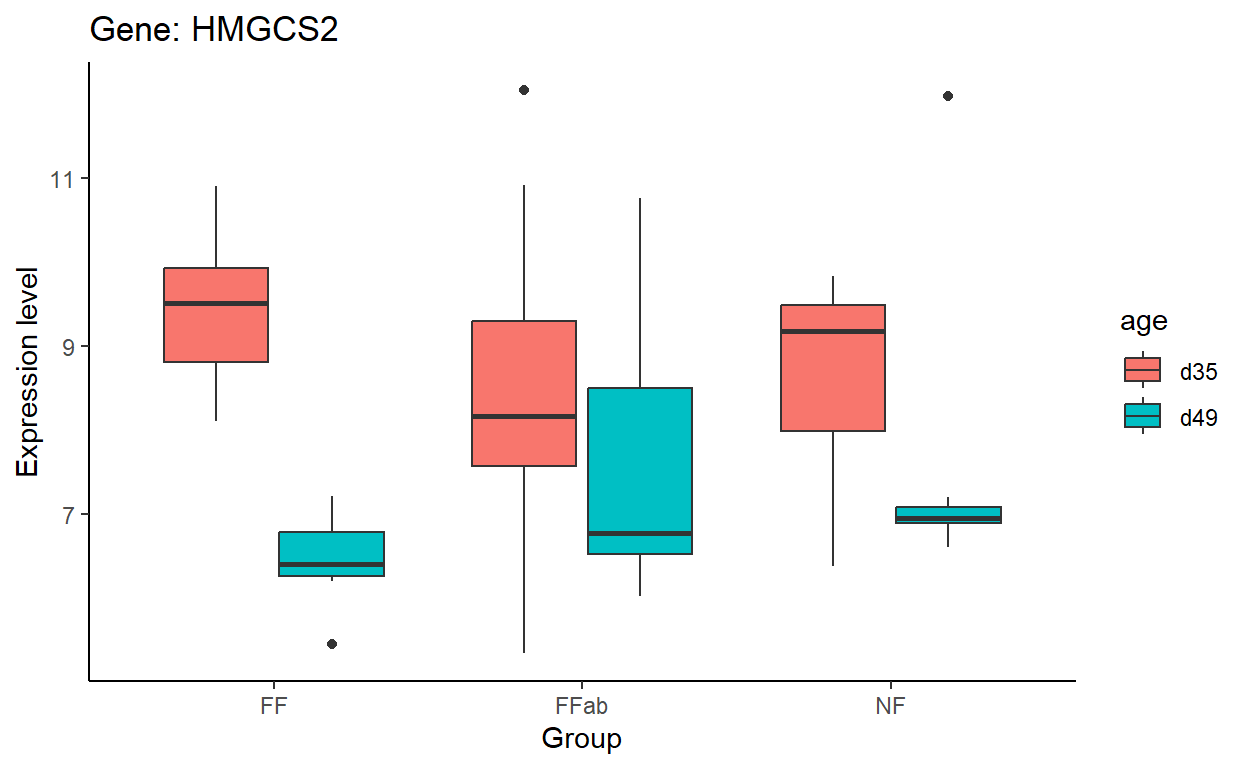 |
| --- | --- |
| 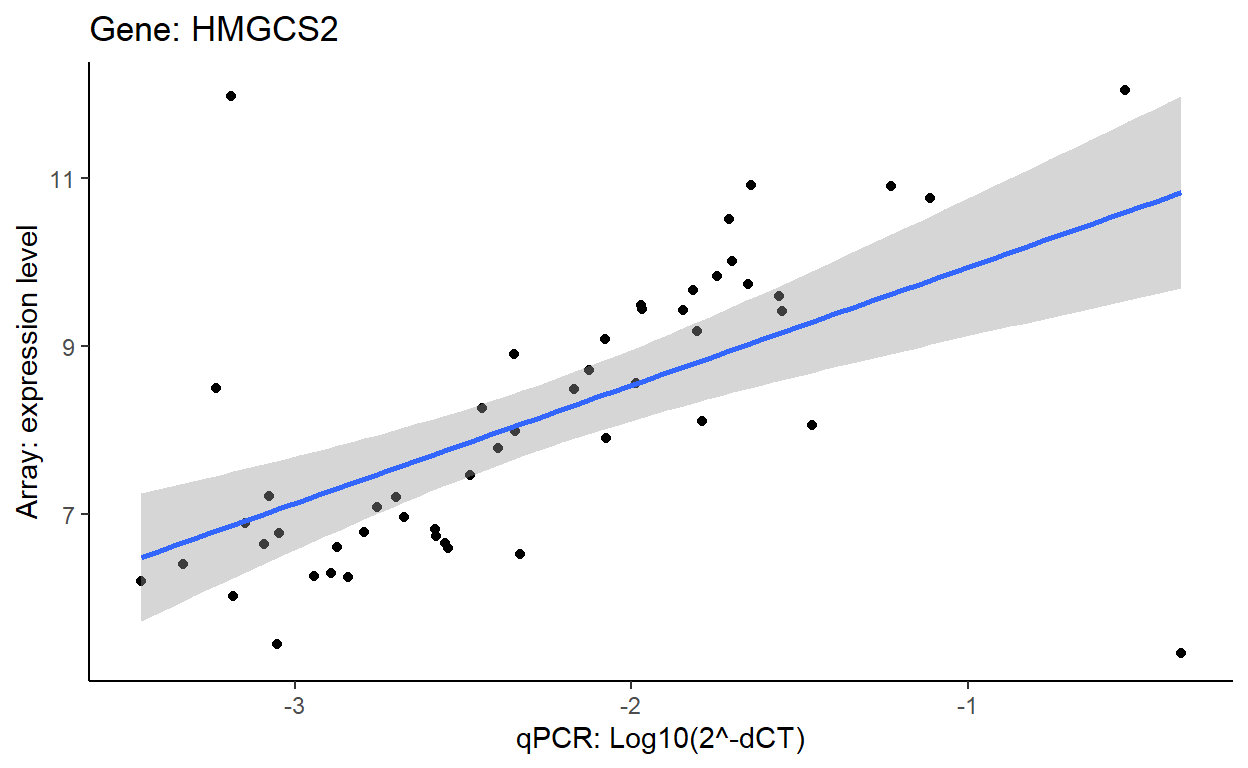 | Spearman's rank correlation rho  p-value = 4.243e-07  rho =0.6553846 |

| 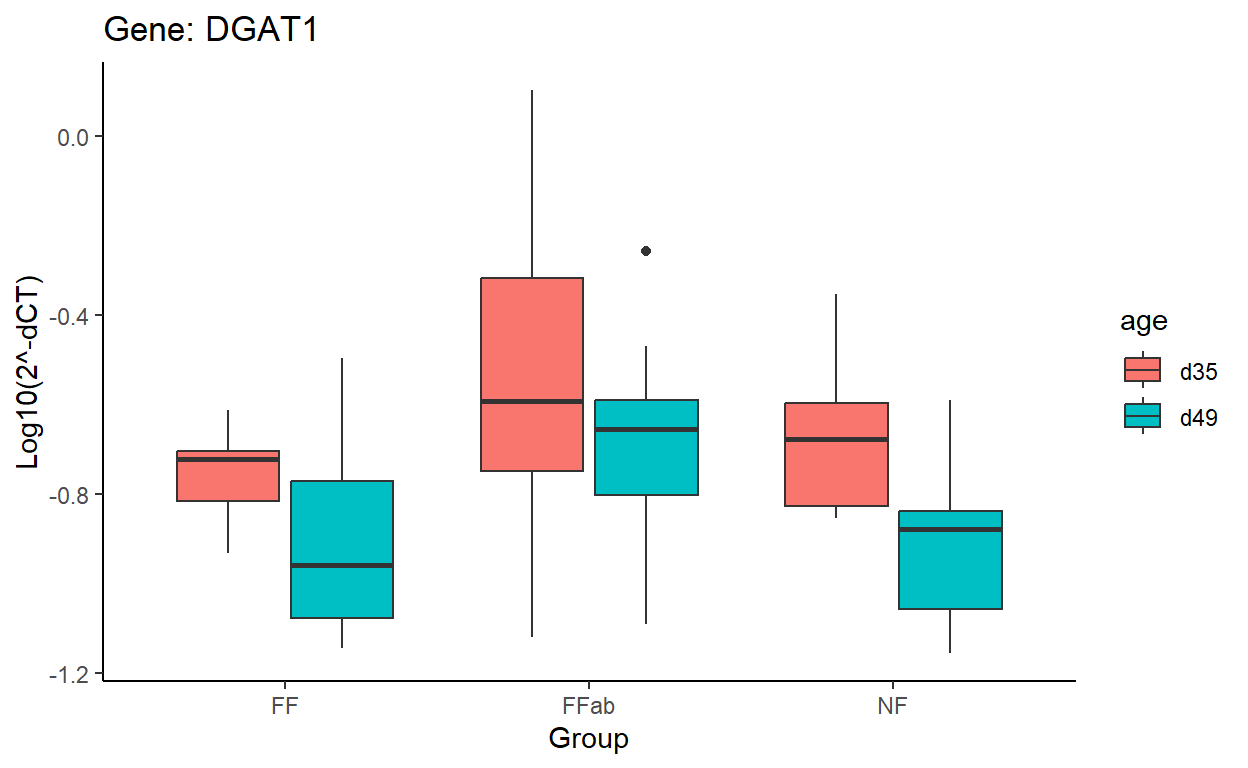 | 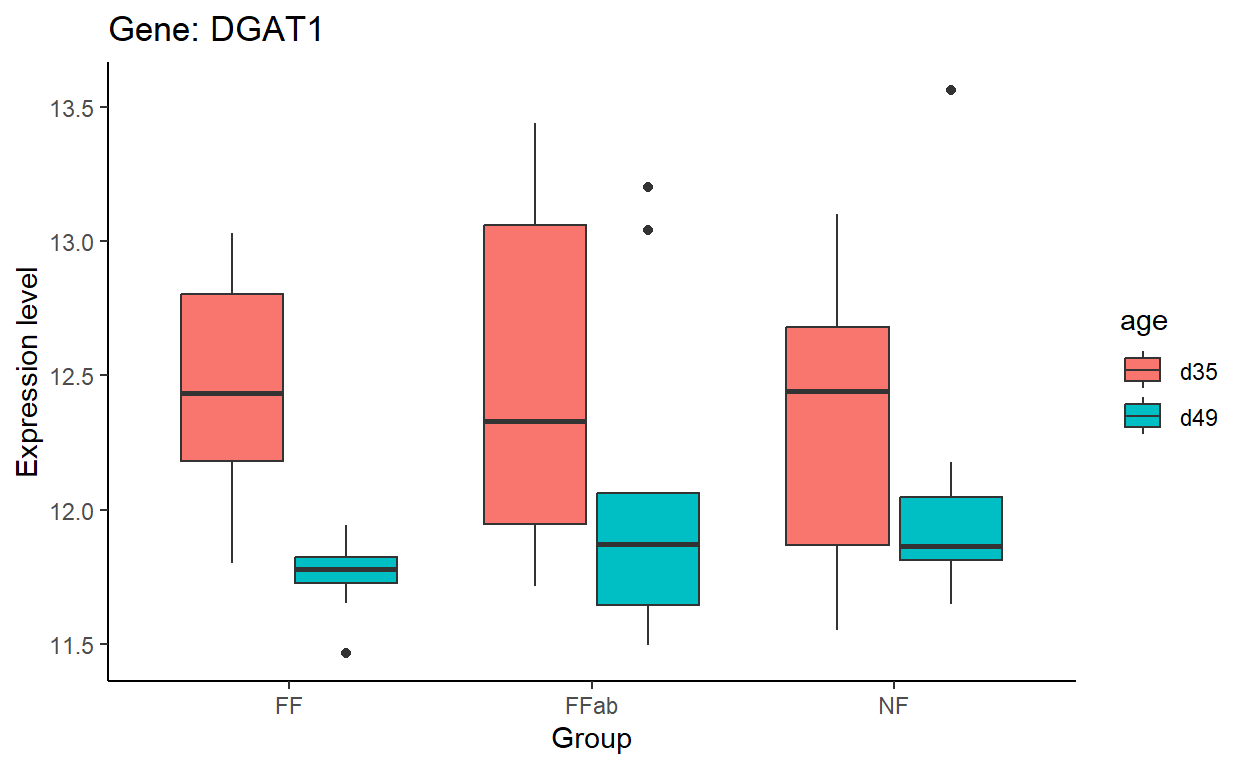 |
| --- | --- |
| 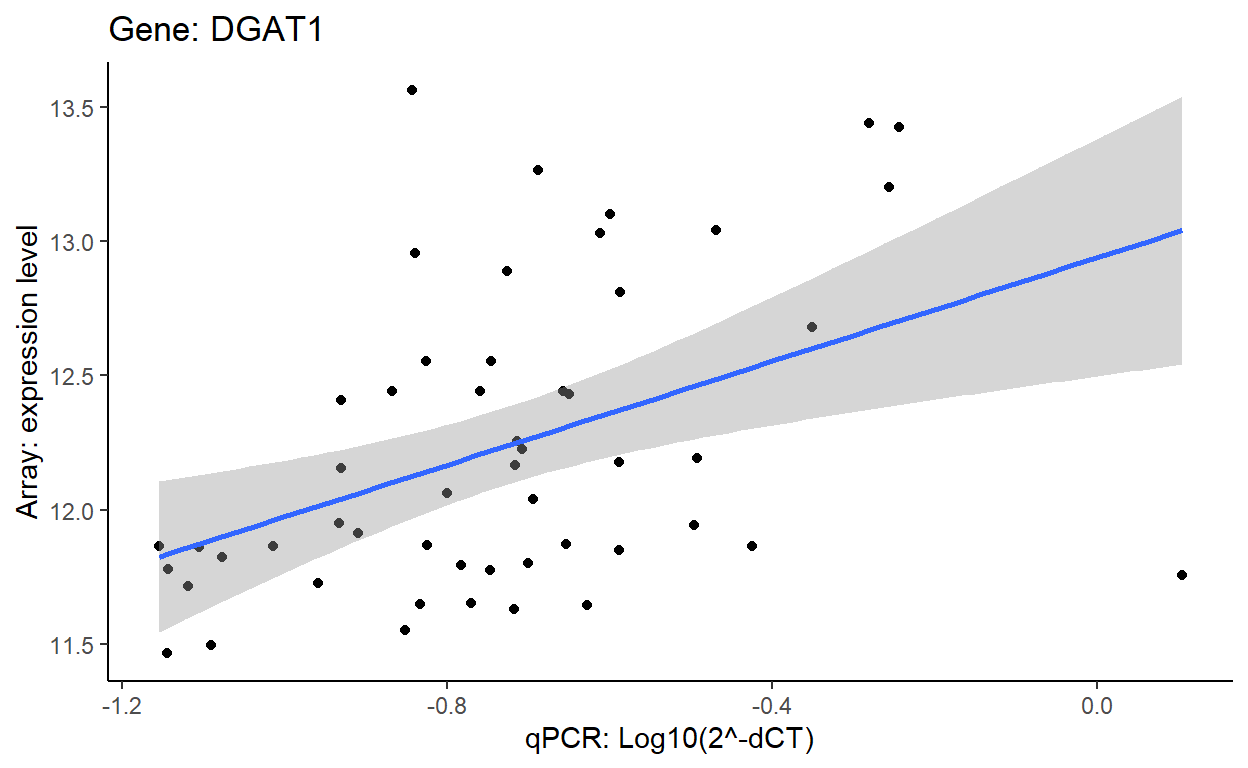 | Spearman's rank correlation rho  p-value = 0.0008744  rho = 0.4517203 |

| 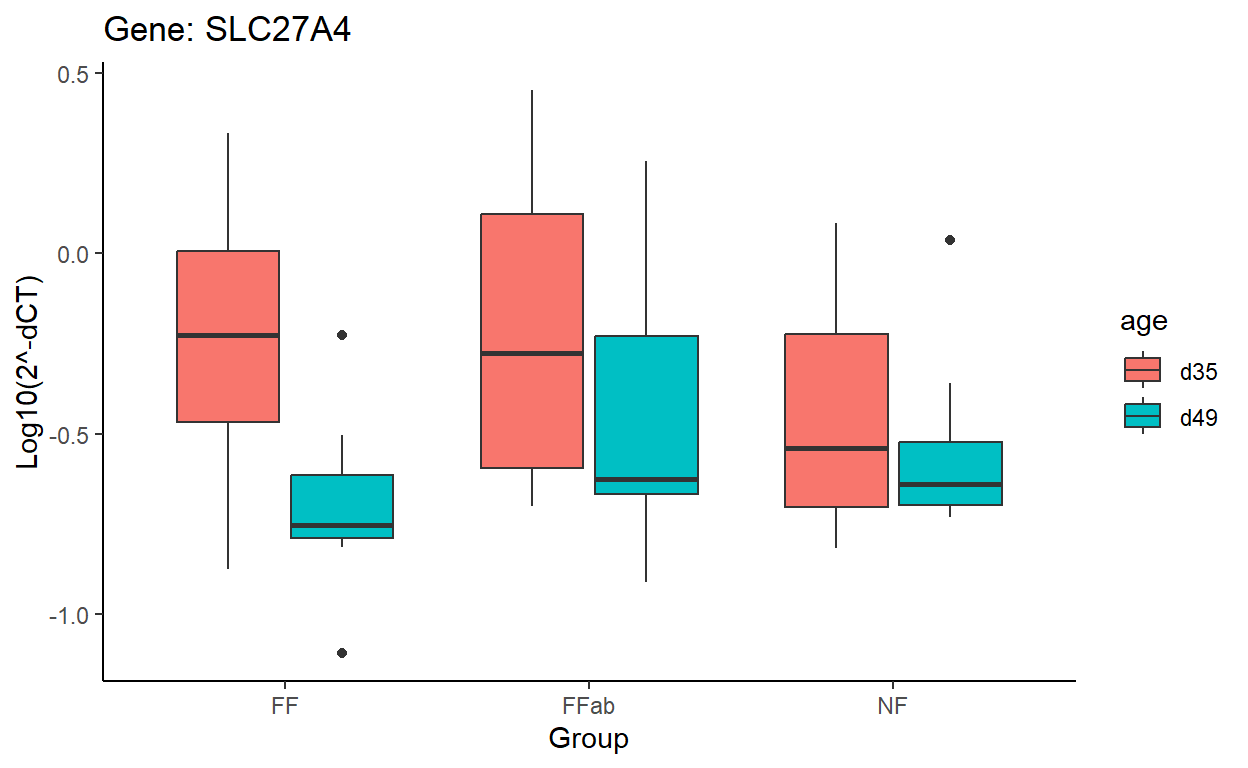 | 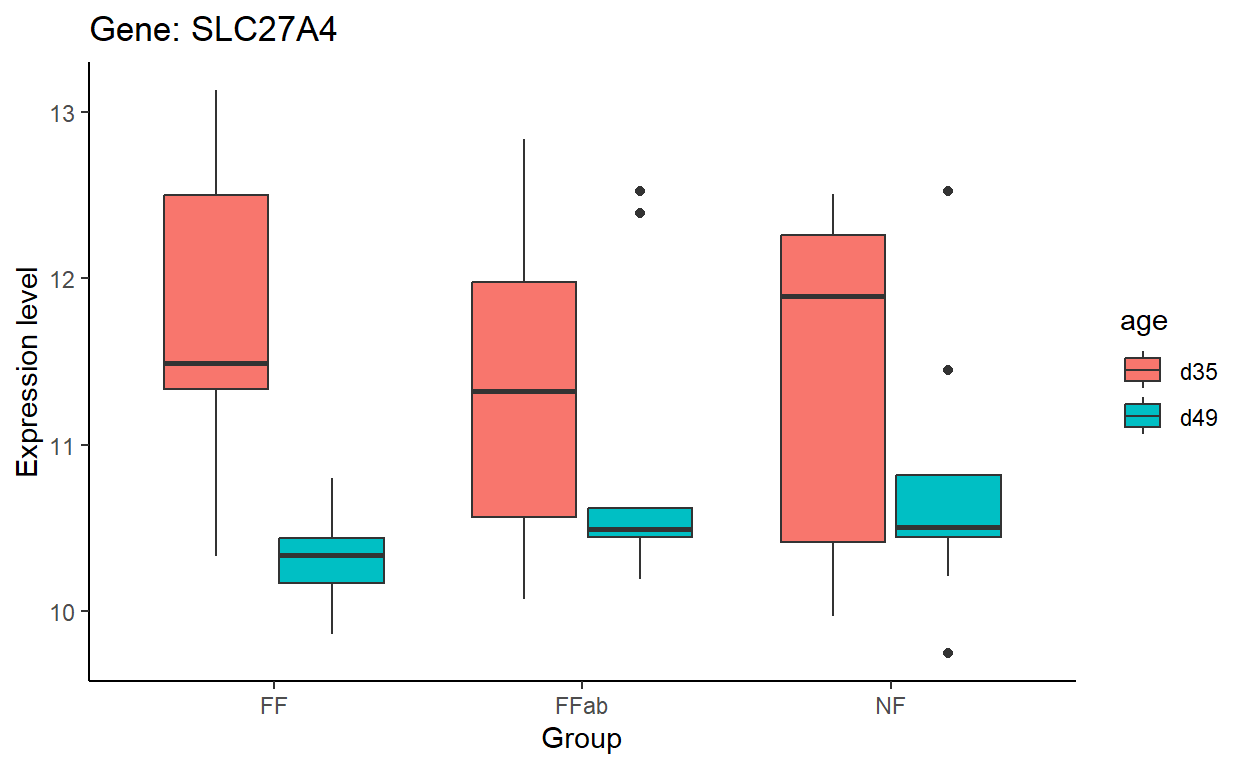 |
| --- | --- |
| 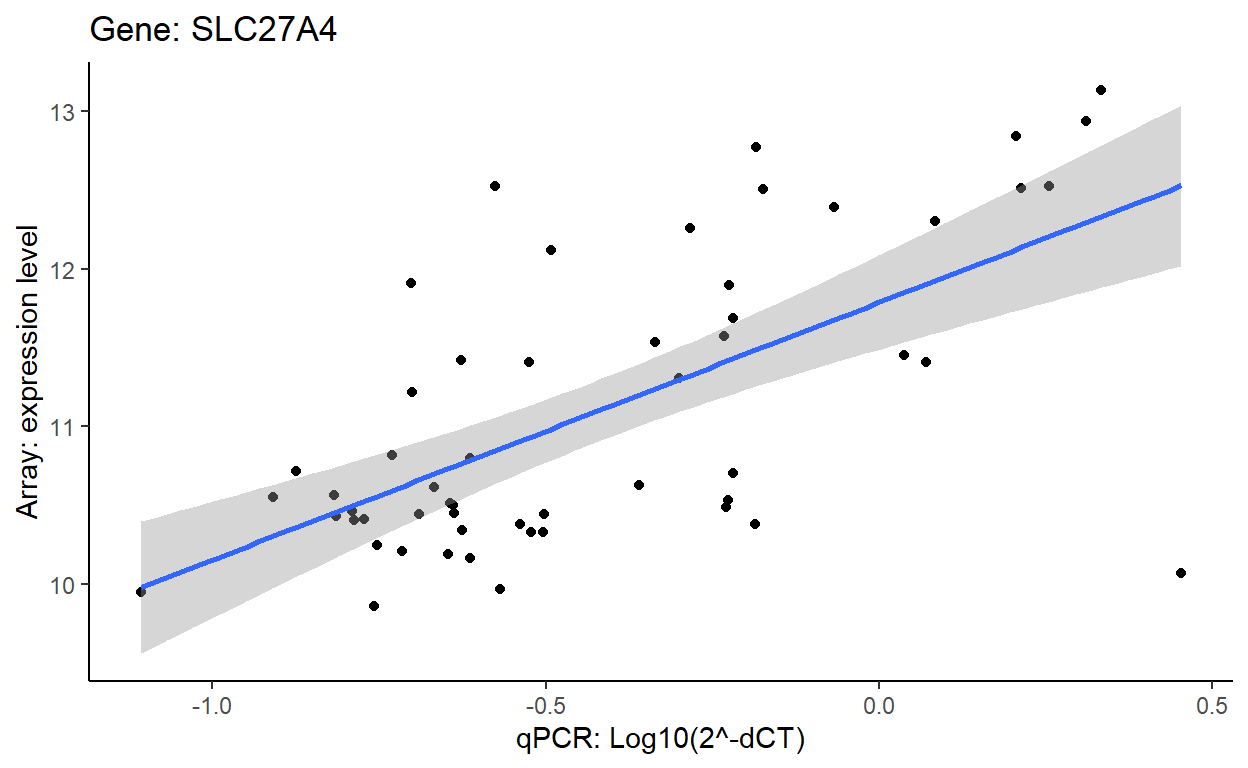 | Spearman's rank correlation rho  p-value = 0.1.338e-05  rho = 0.5585137 |

| 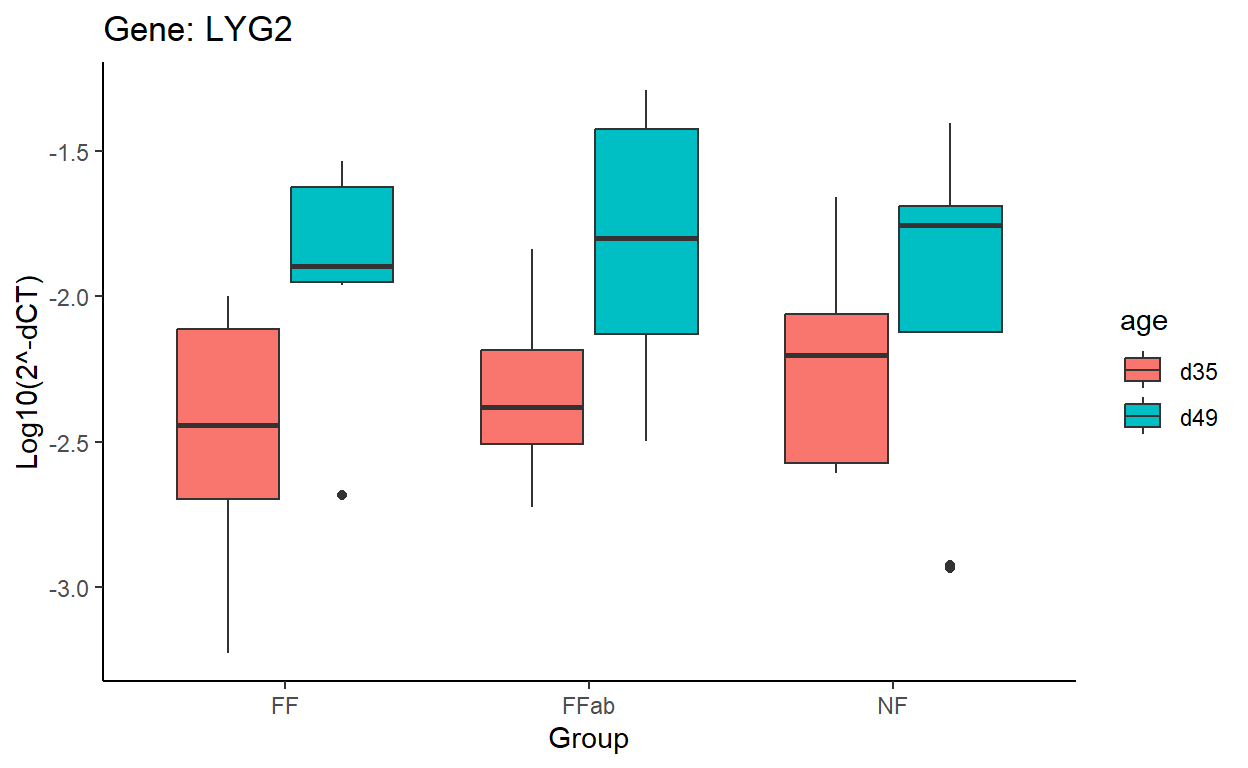 | 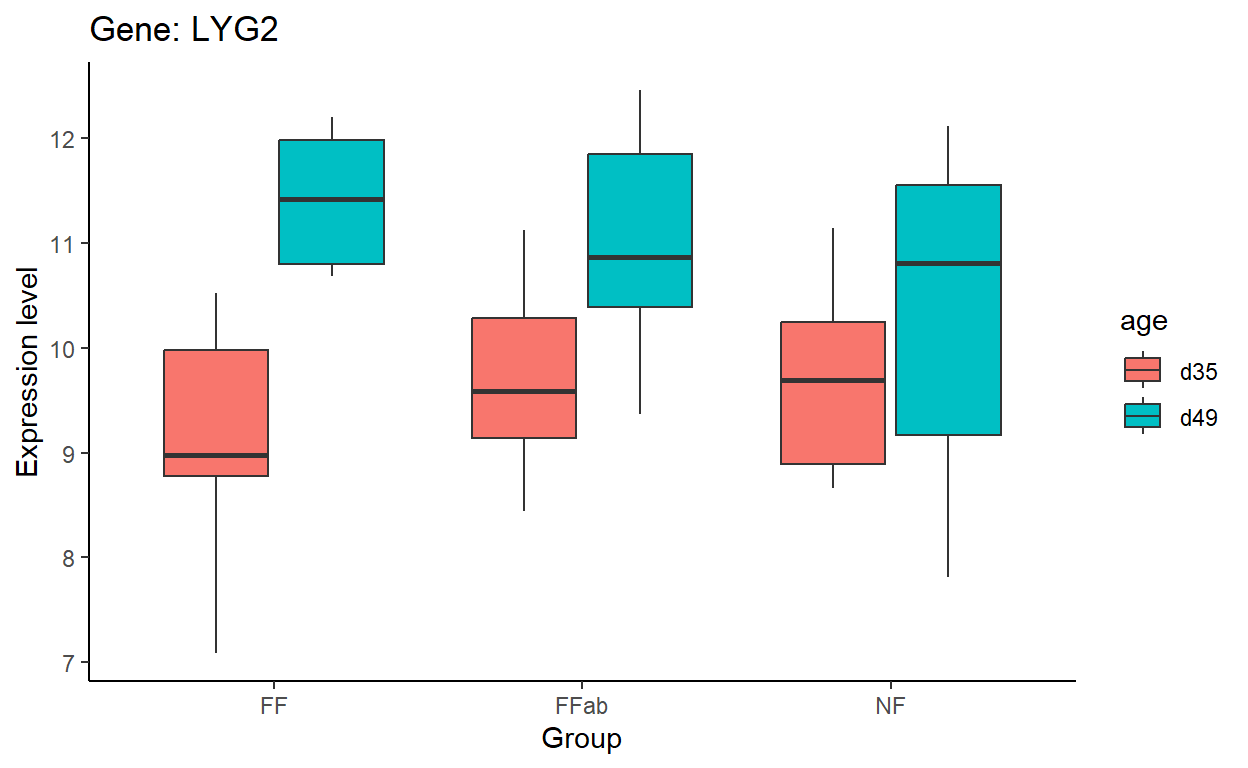 |
| --- | --- |
| 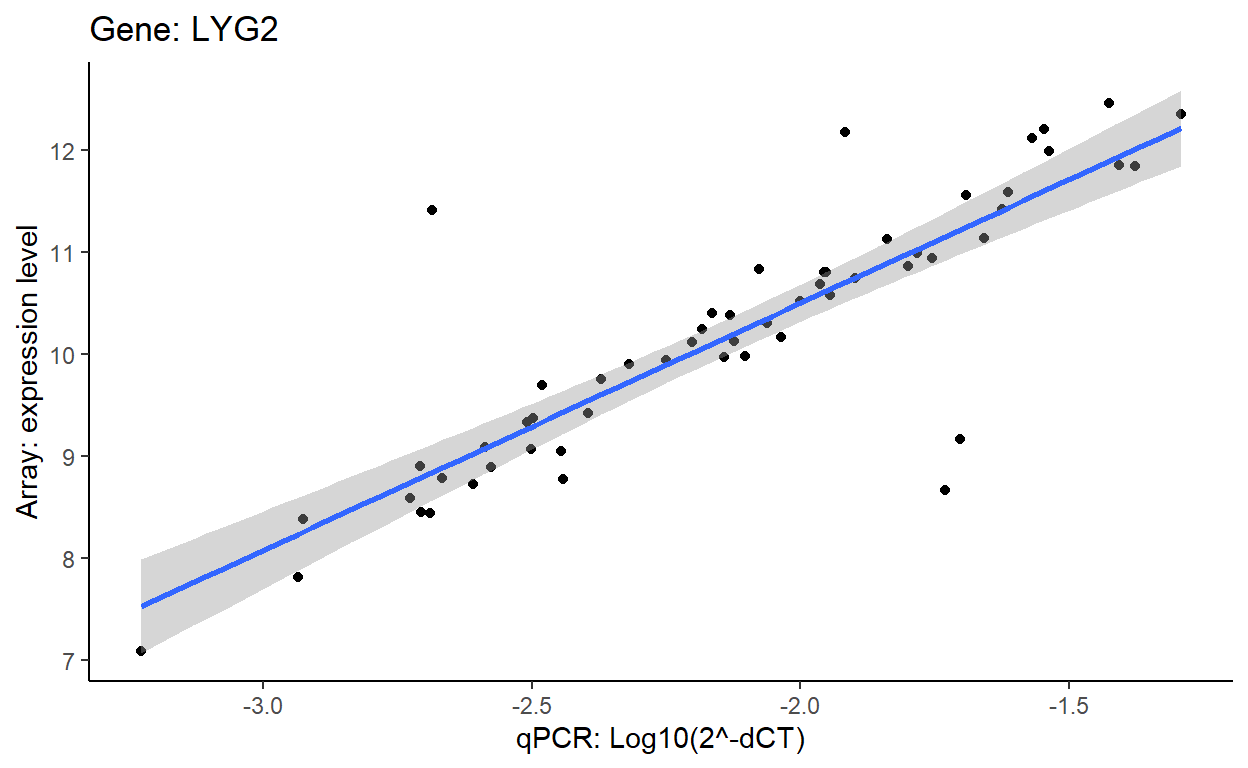 | Spearman's rank correlation rho  p-value = < 2.2e-16  rho = 0.8502392 |

| 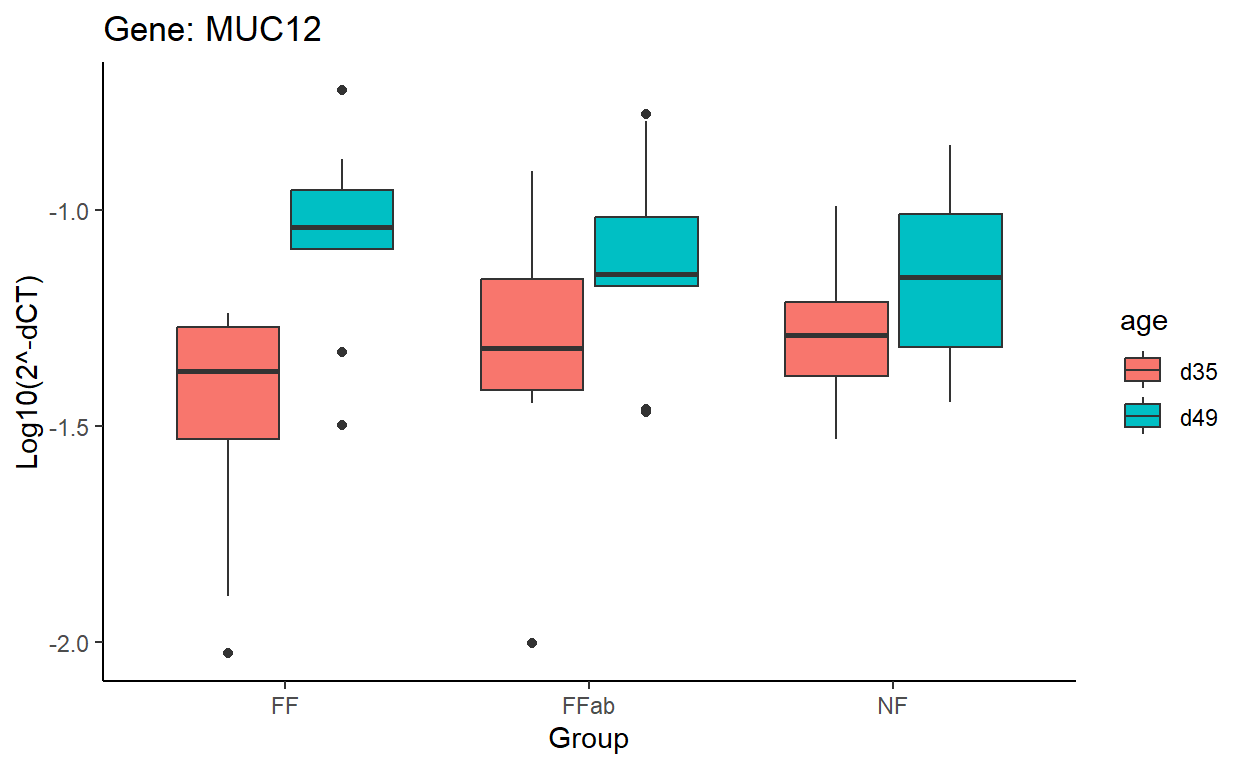 | 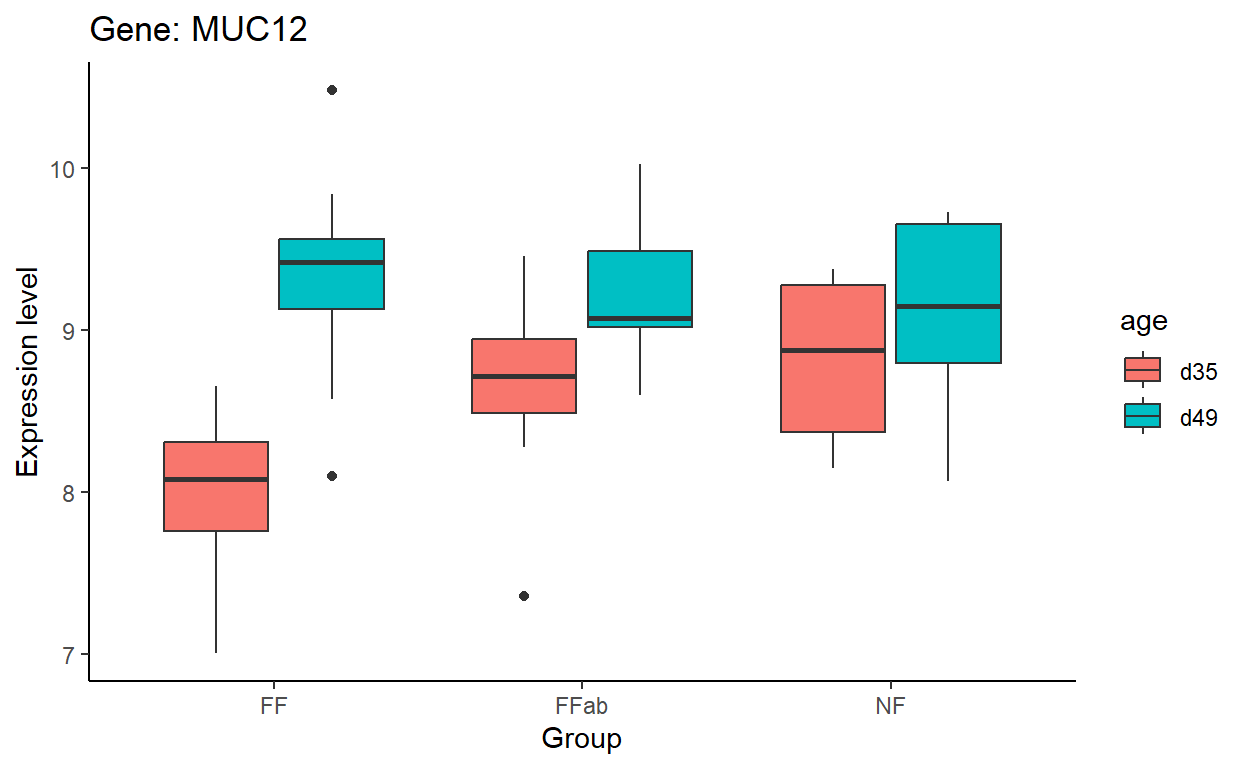 |
| --- | --- |
| 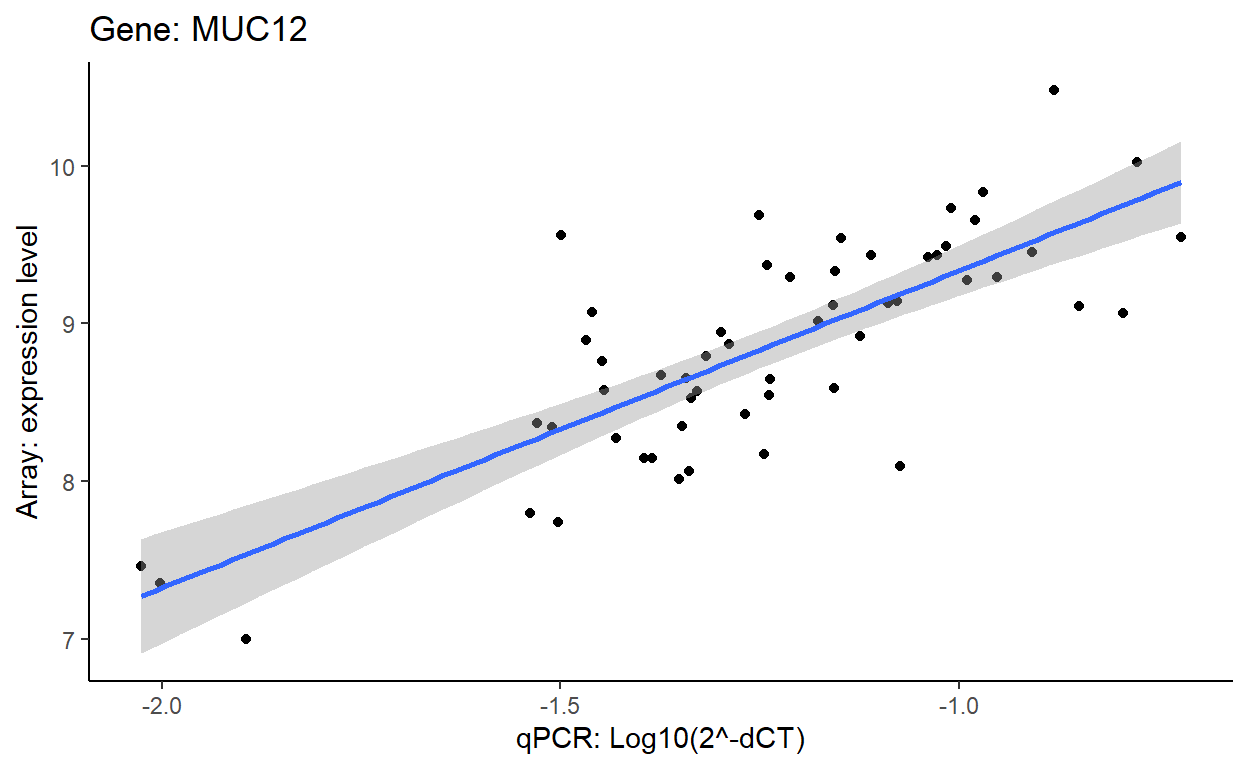 | Spearman's rank correlation rho  p-value = < 2.2e-16  rho = 0.7289815 |

| 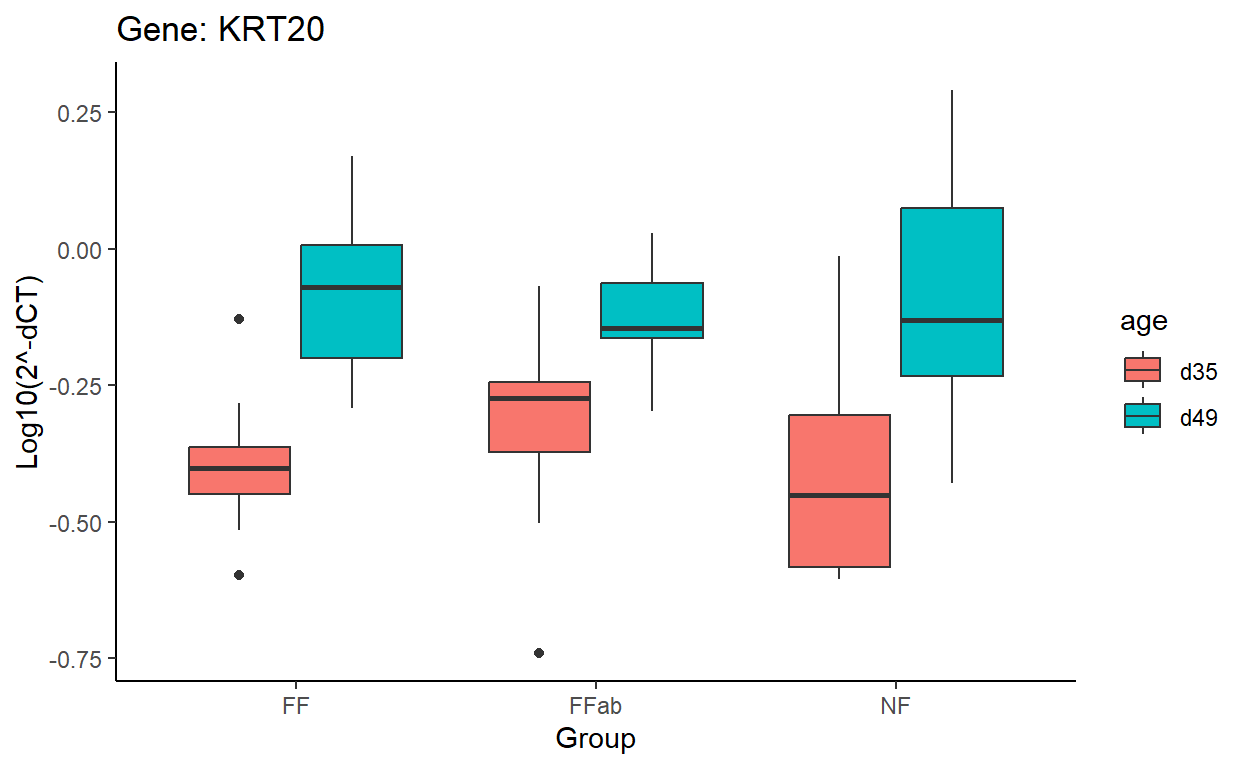 | 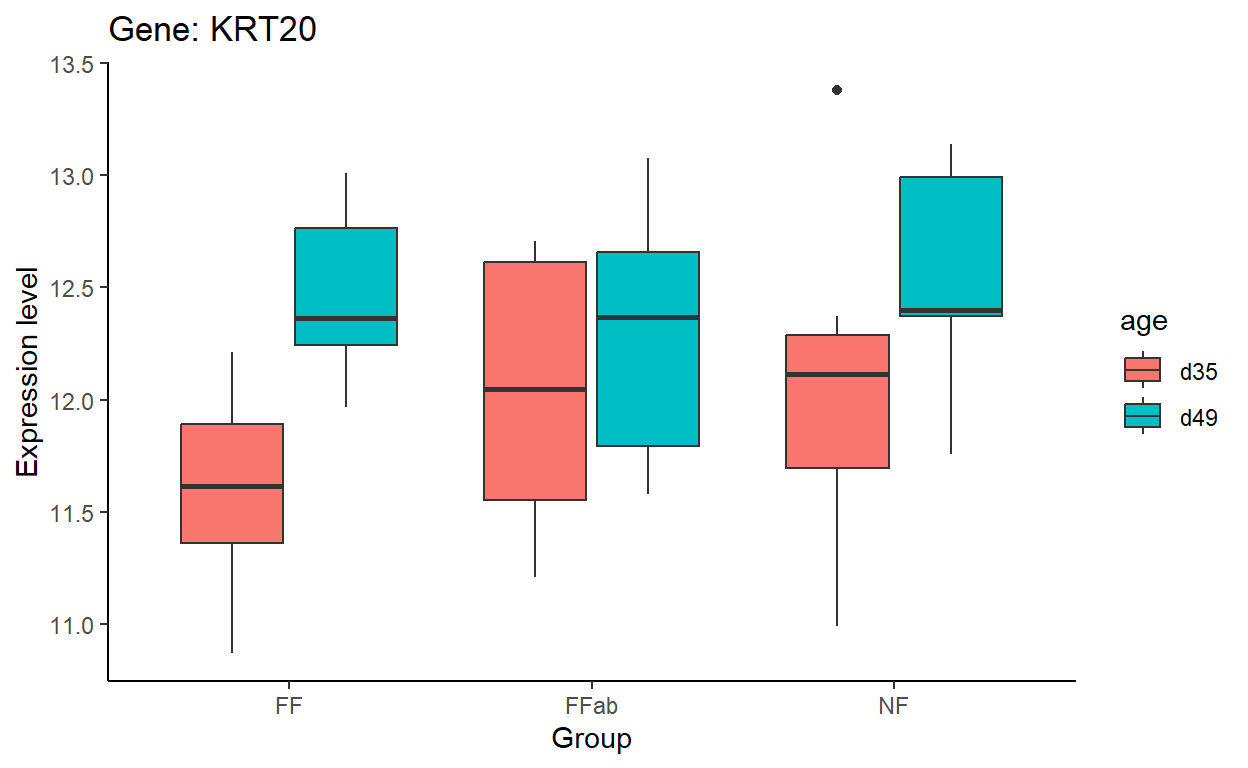 |
| --- | --- |
| 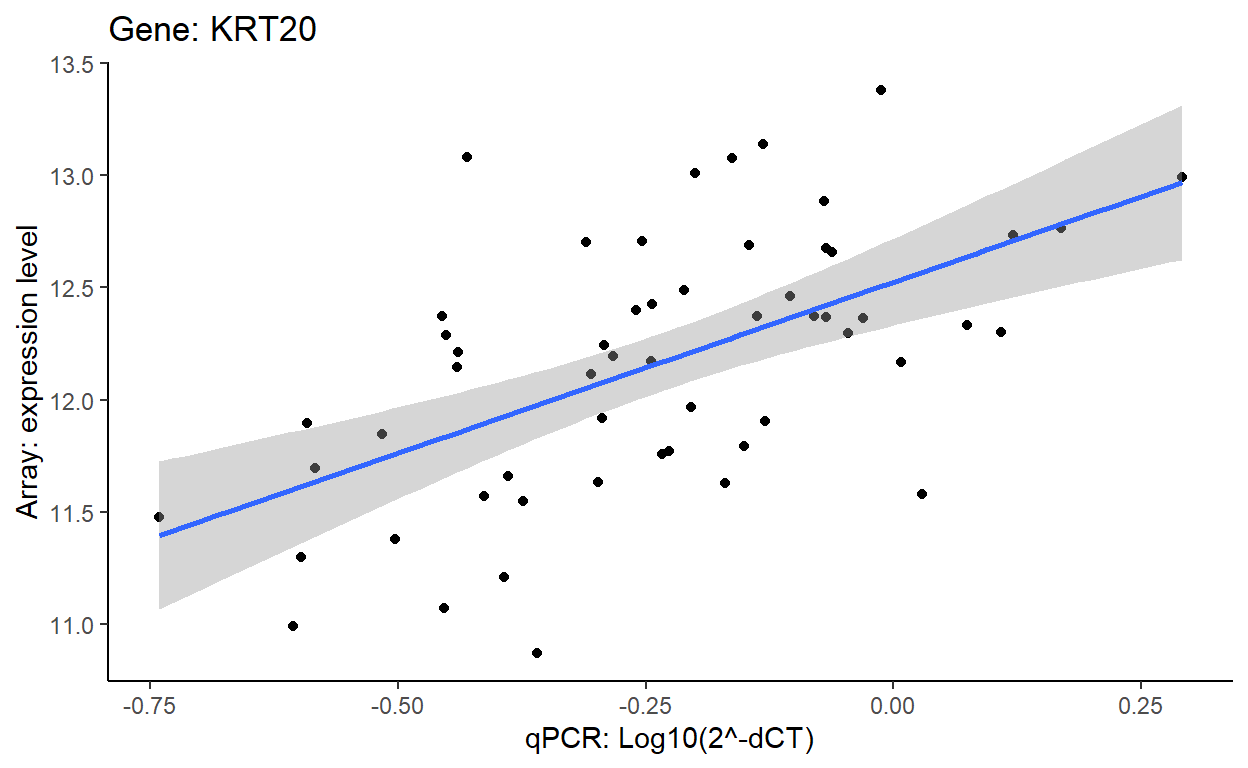 | Spearman's rank correlation rho  p-value = 6.974e-06  rho = 0.5607068 |

| 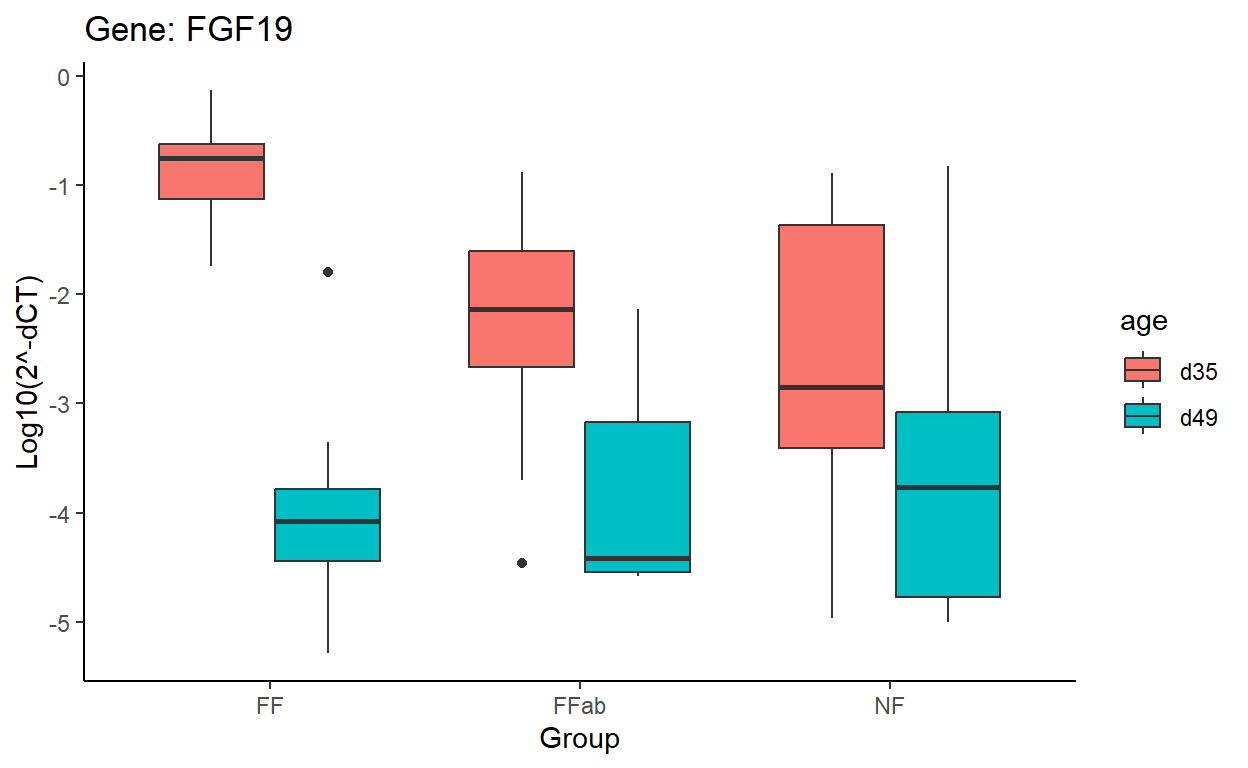 | 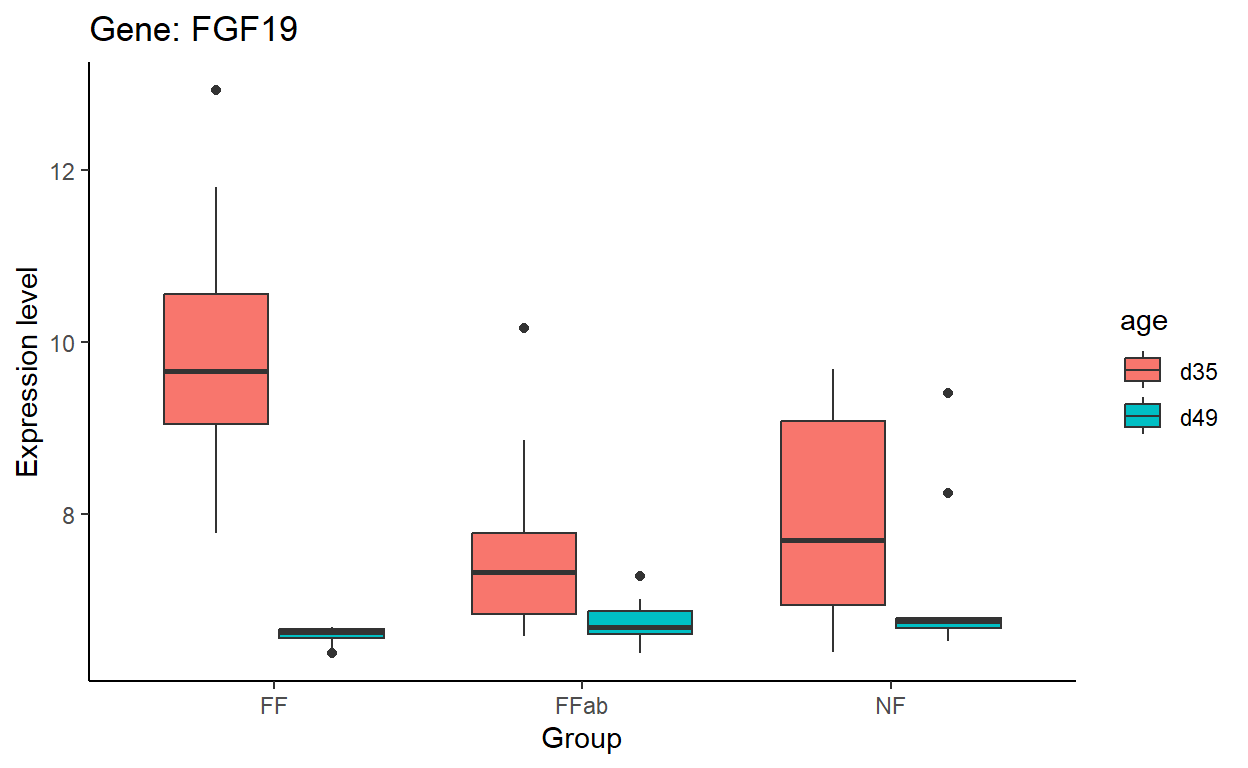 |
| --- | --- |
| 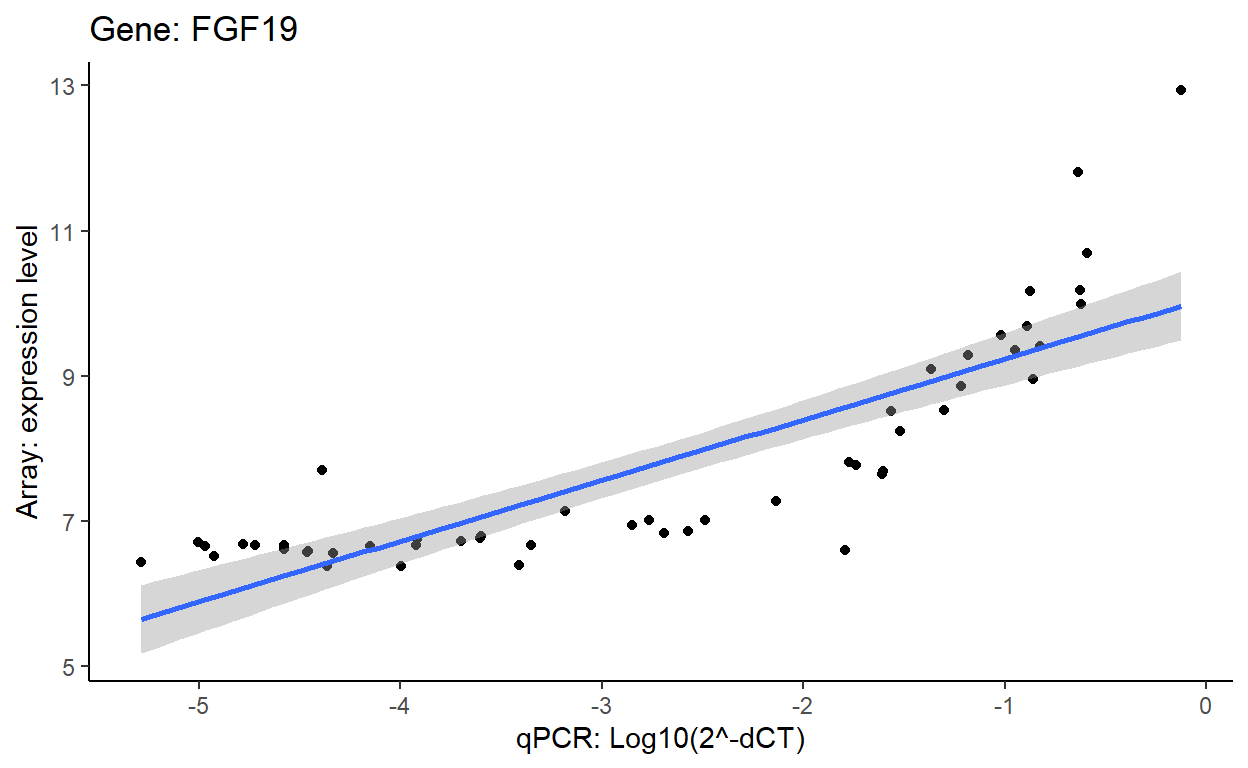 | Spearman's rank correlation rho  p-value = < 2.2e-16  rho = 0.8914027 |

| 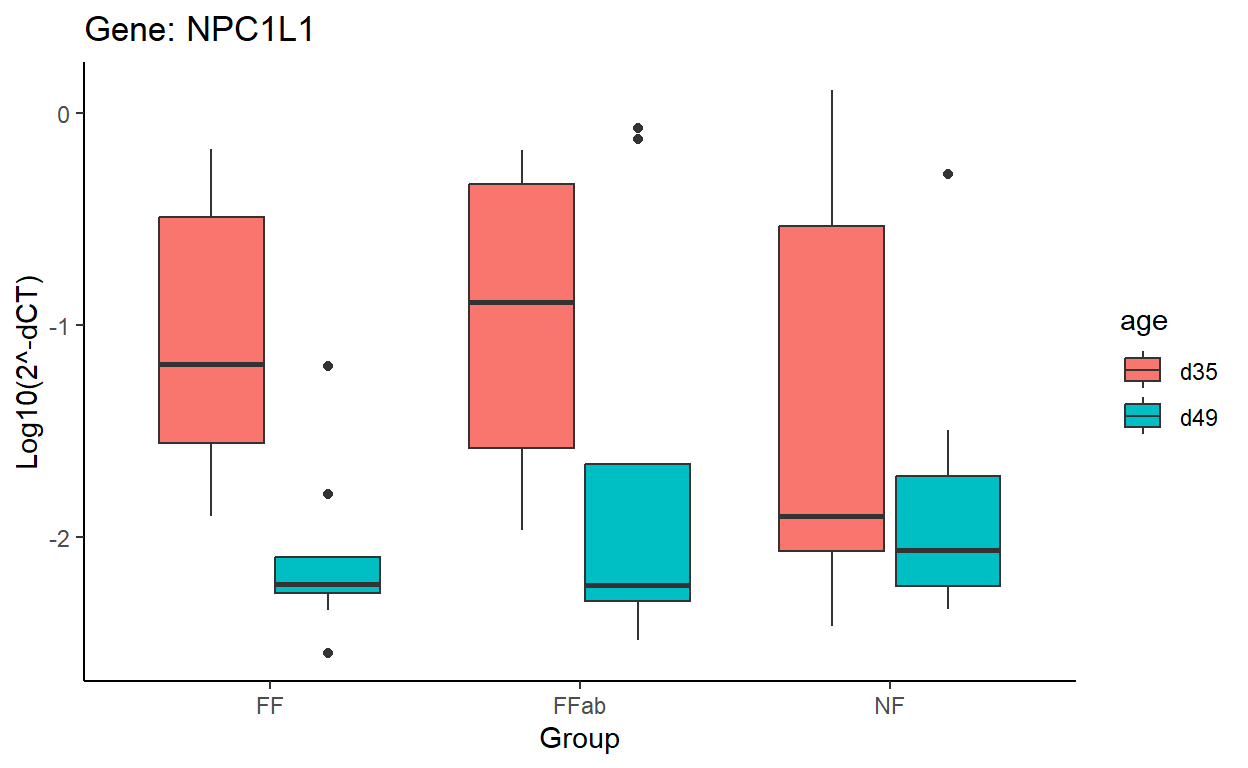 | 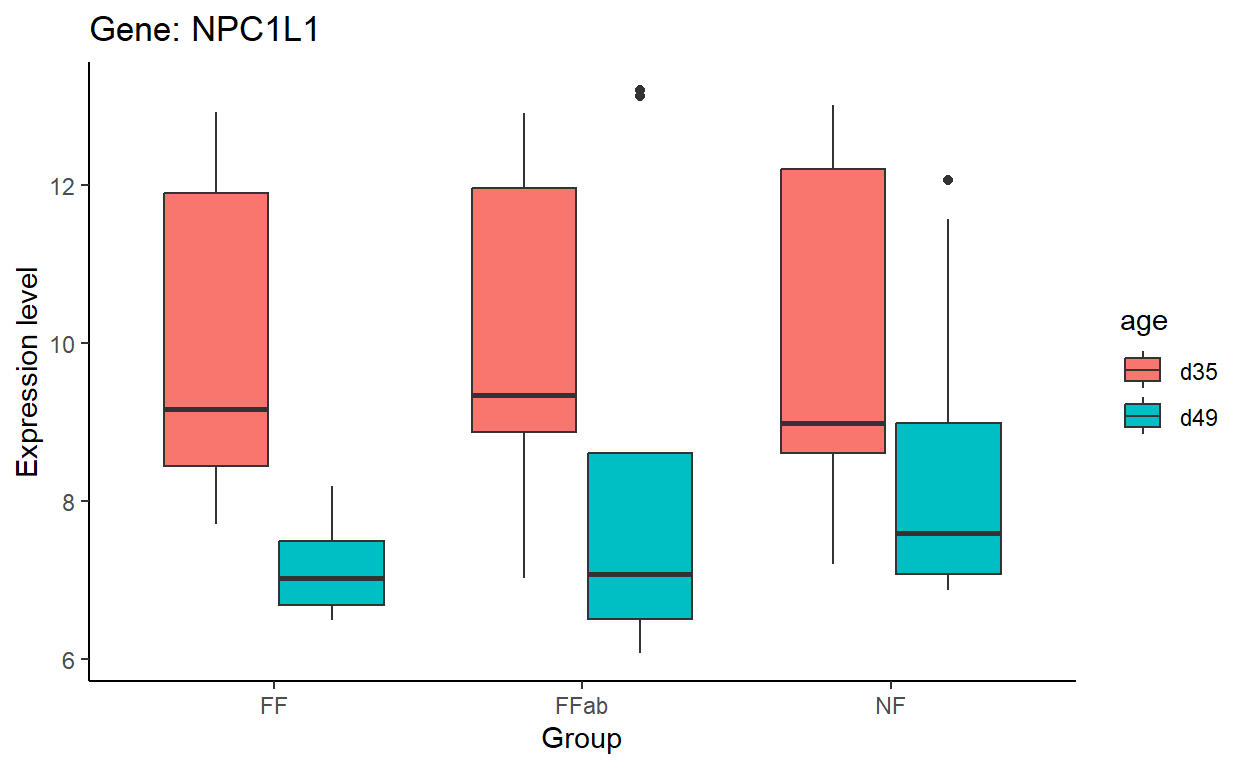 |
| --- | --- |
| 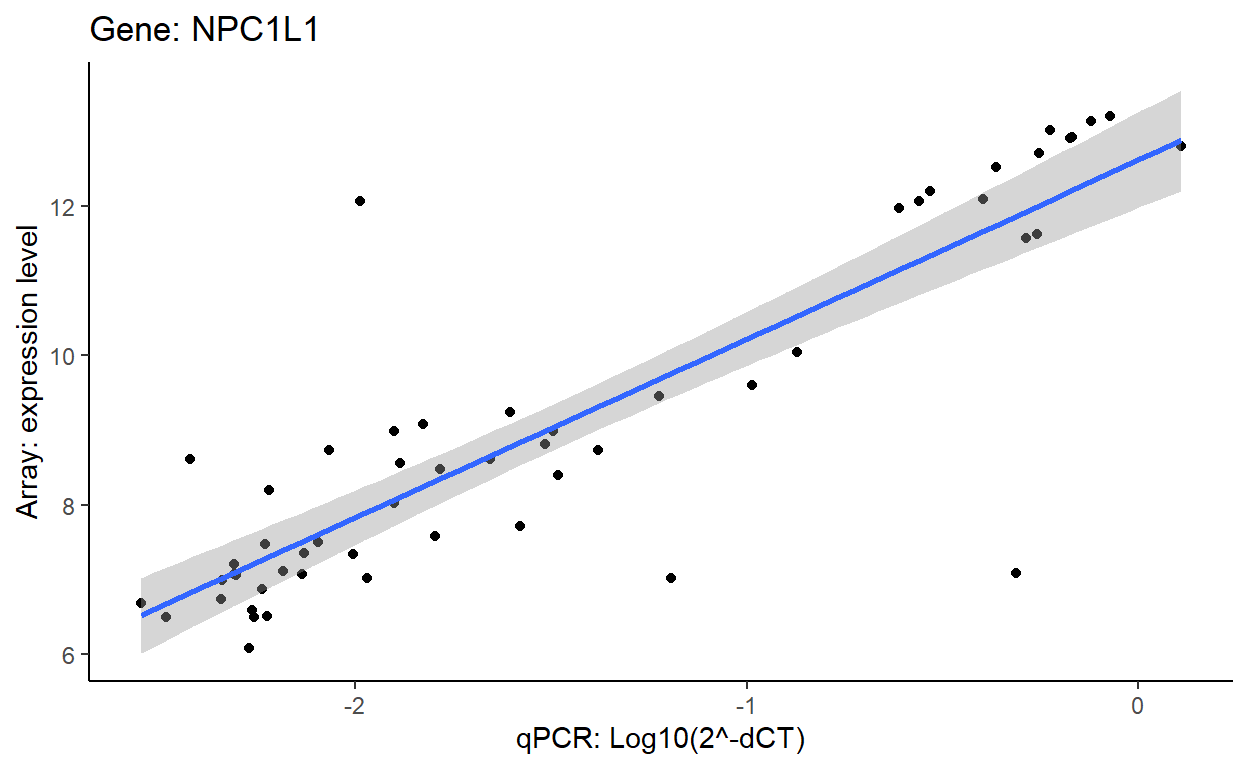 | Spearman's rank correlation rho  p-value = < 2.2e-16  rho = 0.8375902 |

| 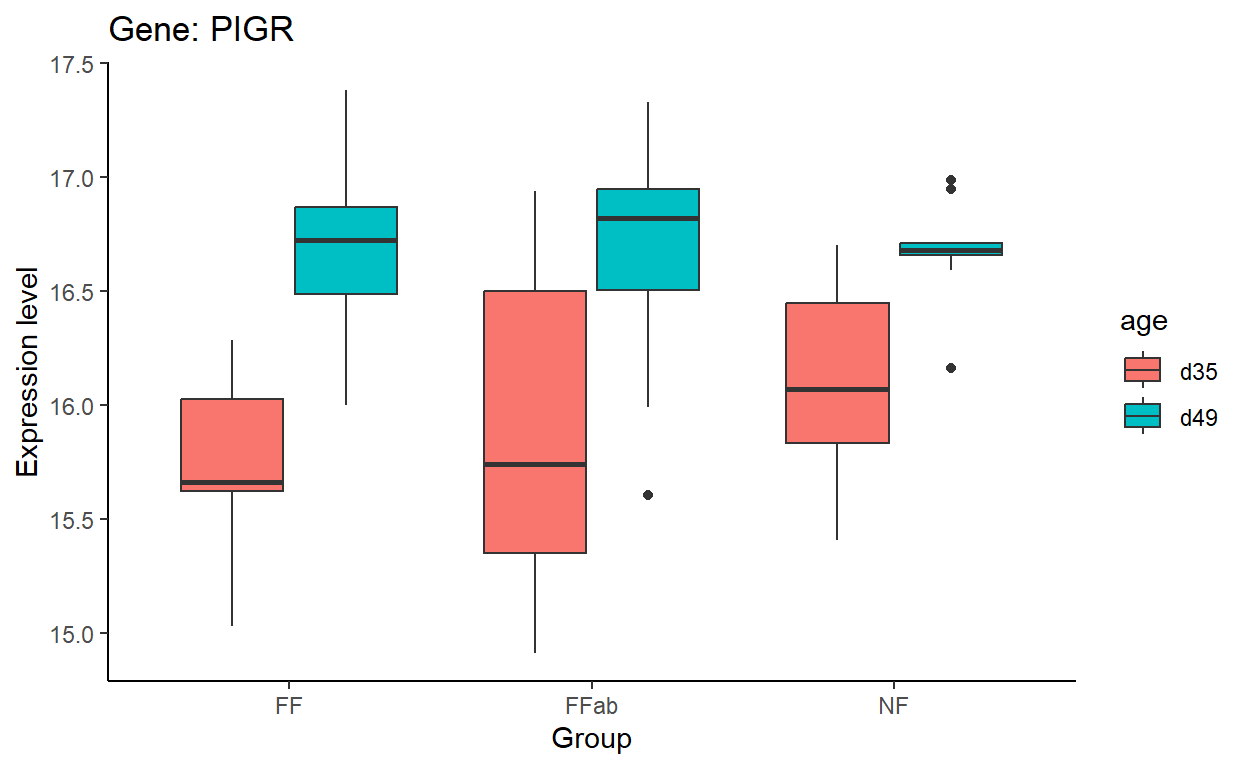 | 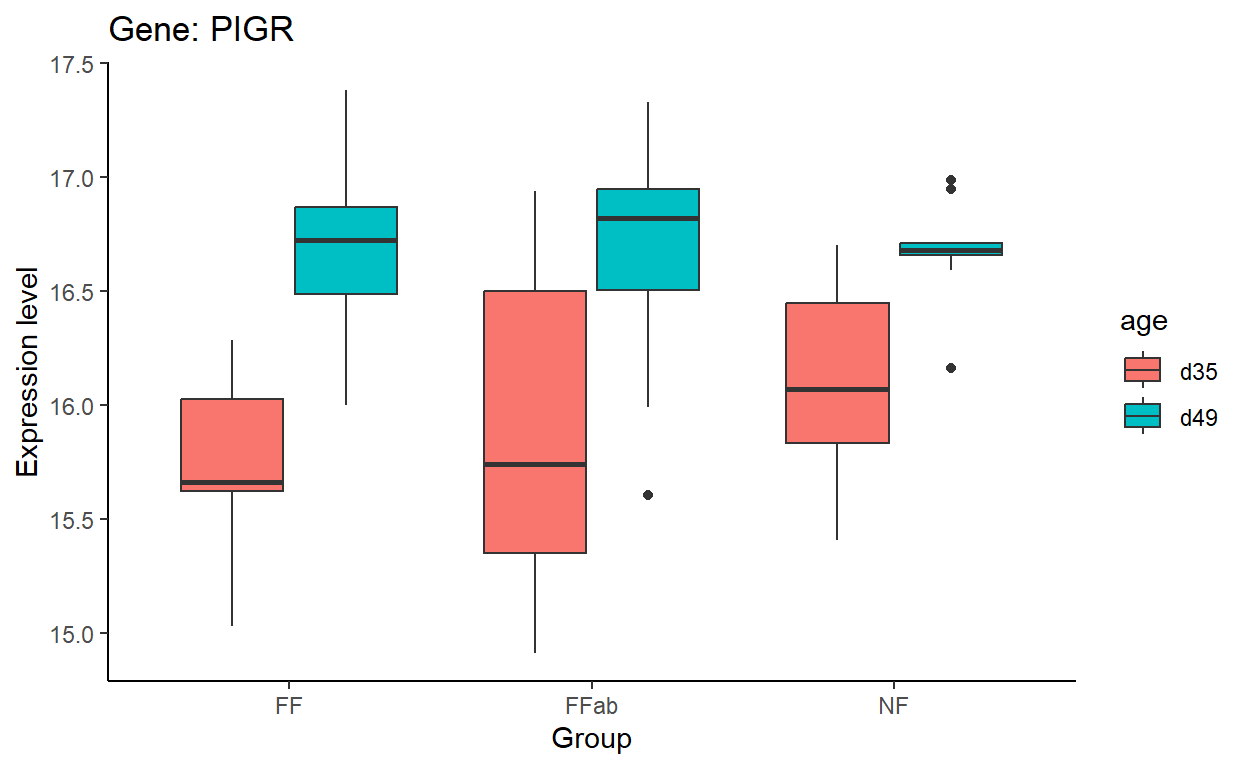 |
| --- | --- |
| 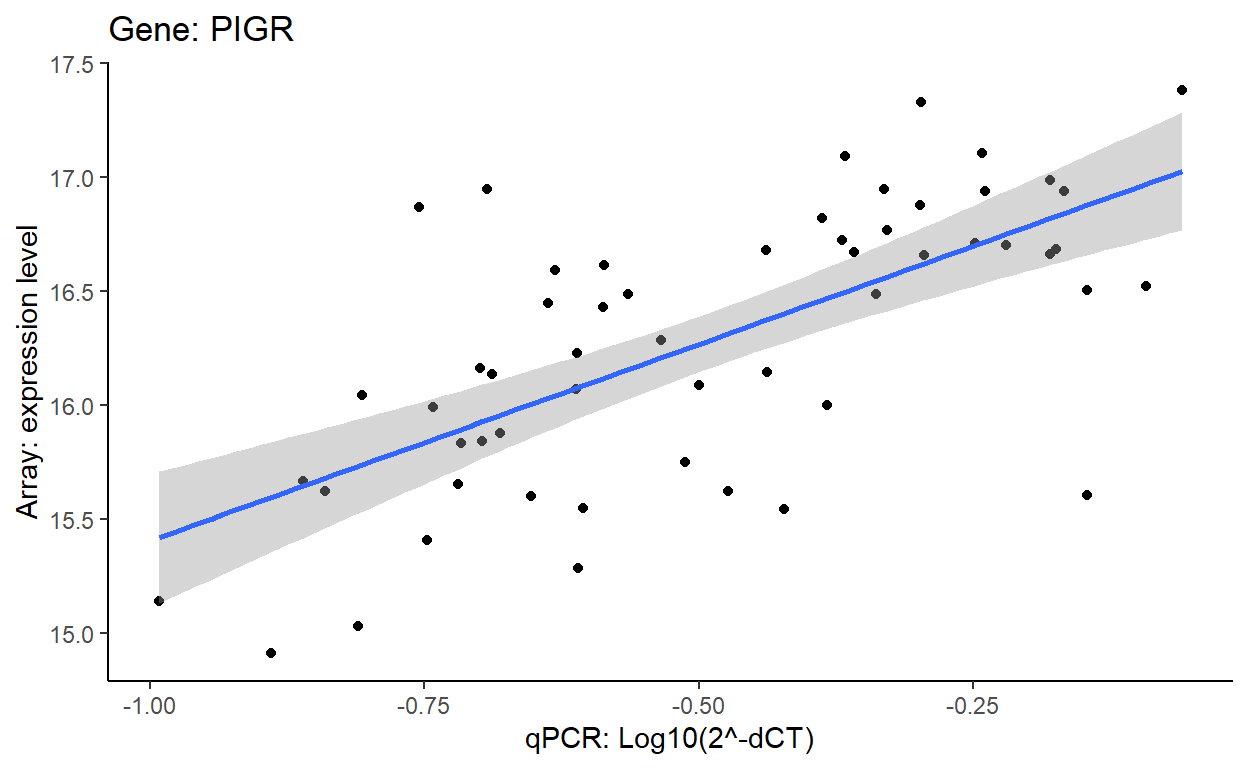 | Spearman's rank correlation rho  p-value = 1.538e-07  rho = 0.6342197 |

| 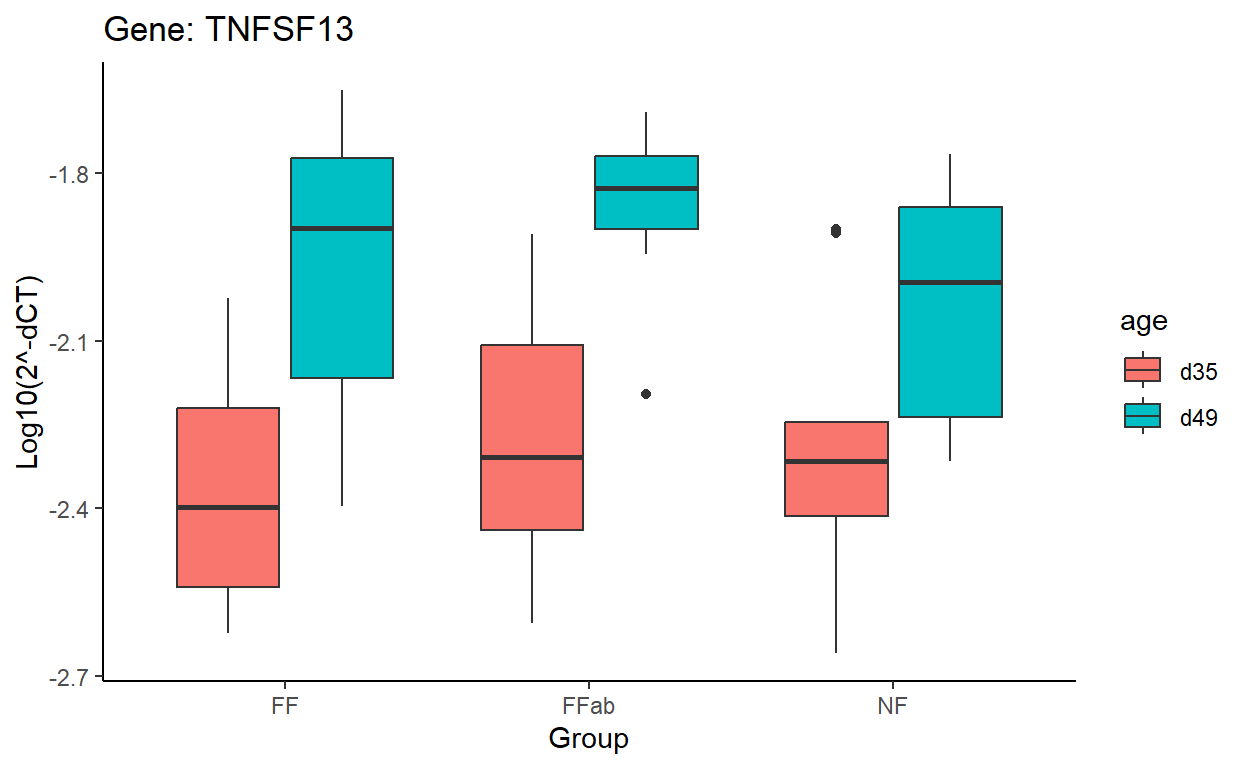 | 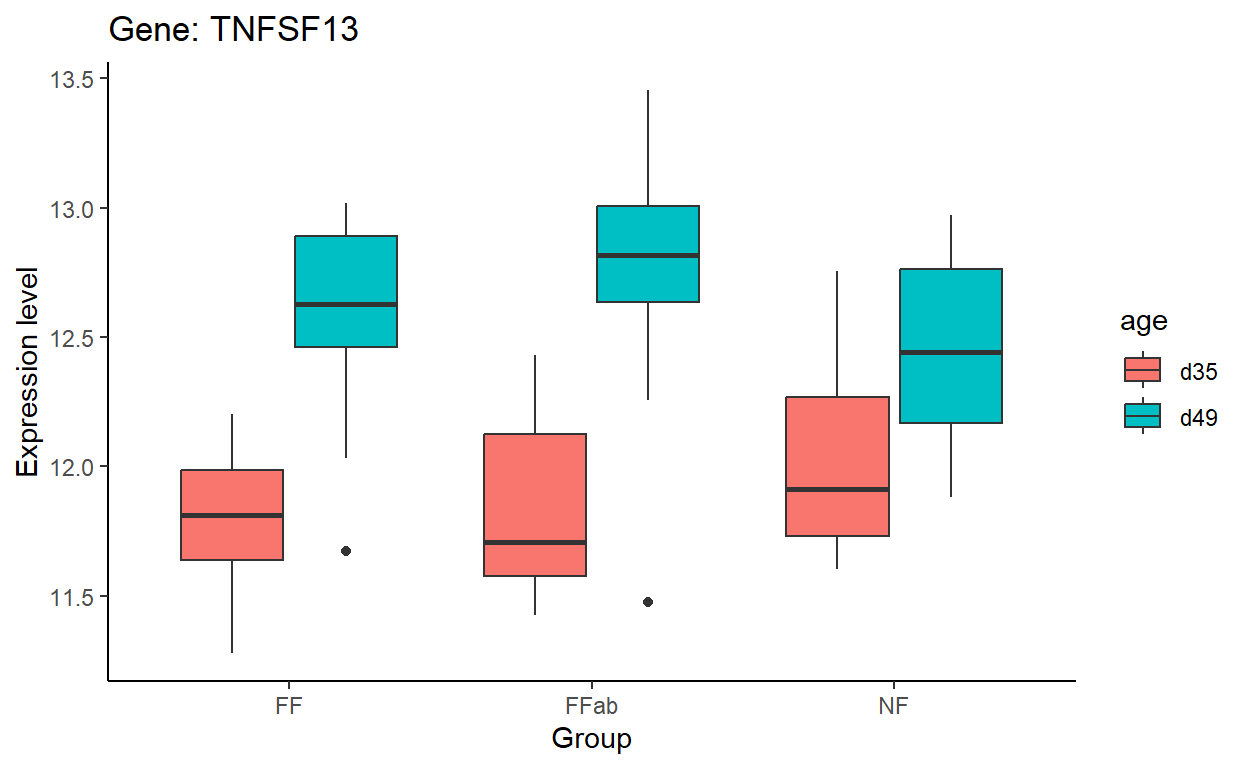 |
| --- | --- |
| 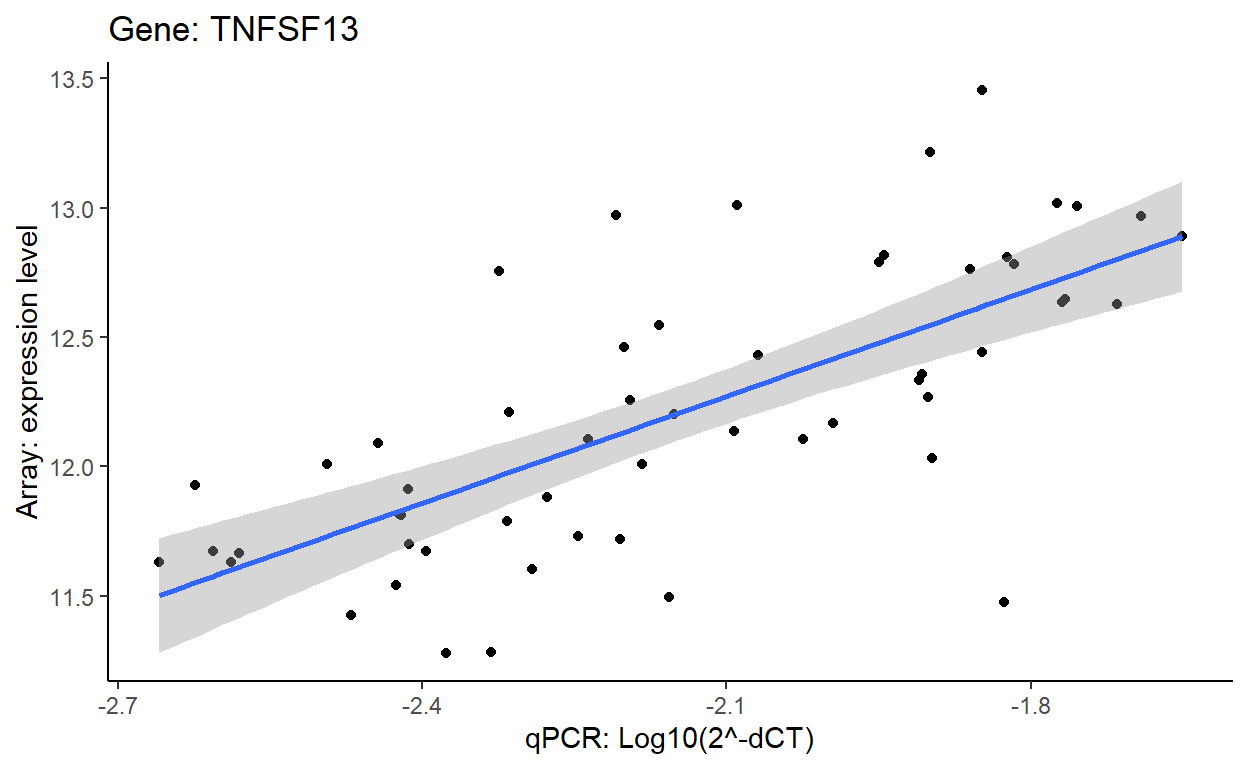 | Spearman's rank correlation rho  p-value = 7.537e-10  rho = 0.7121448 |

| 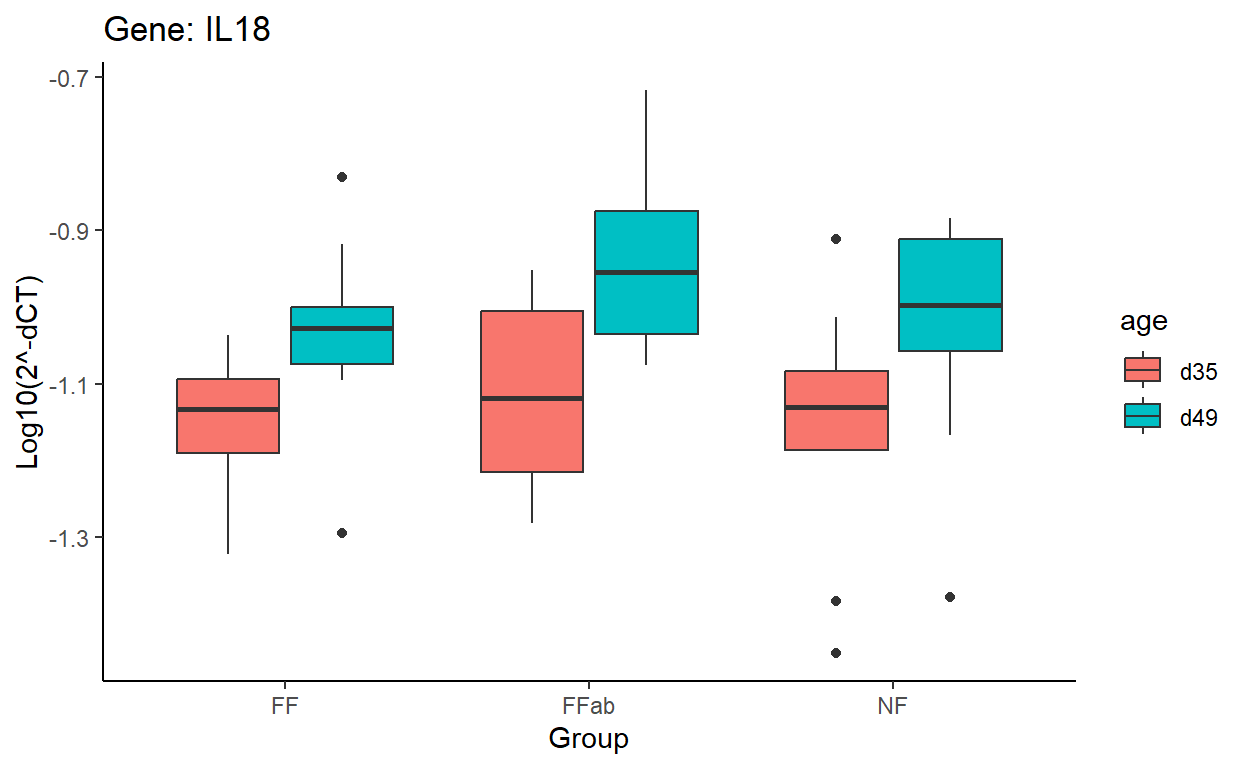 | 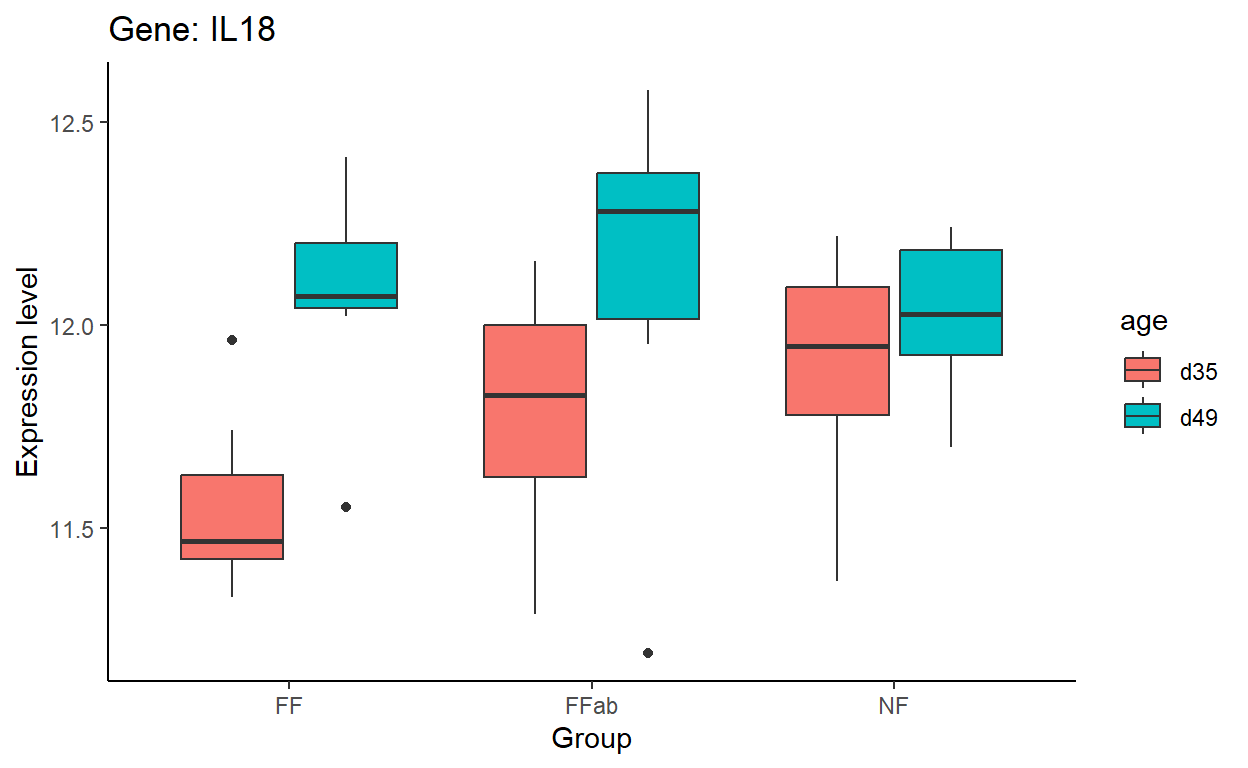 |
| --- | --- |
| 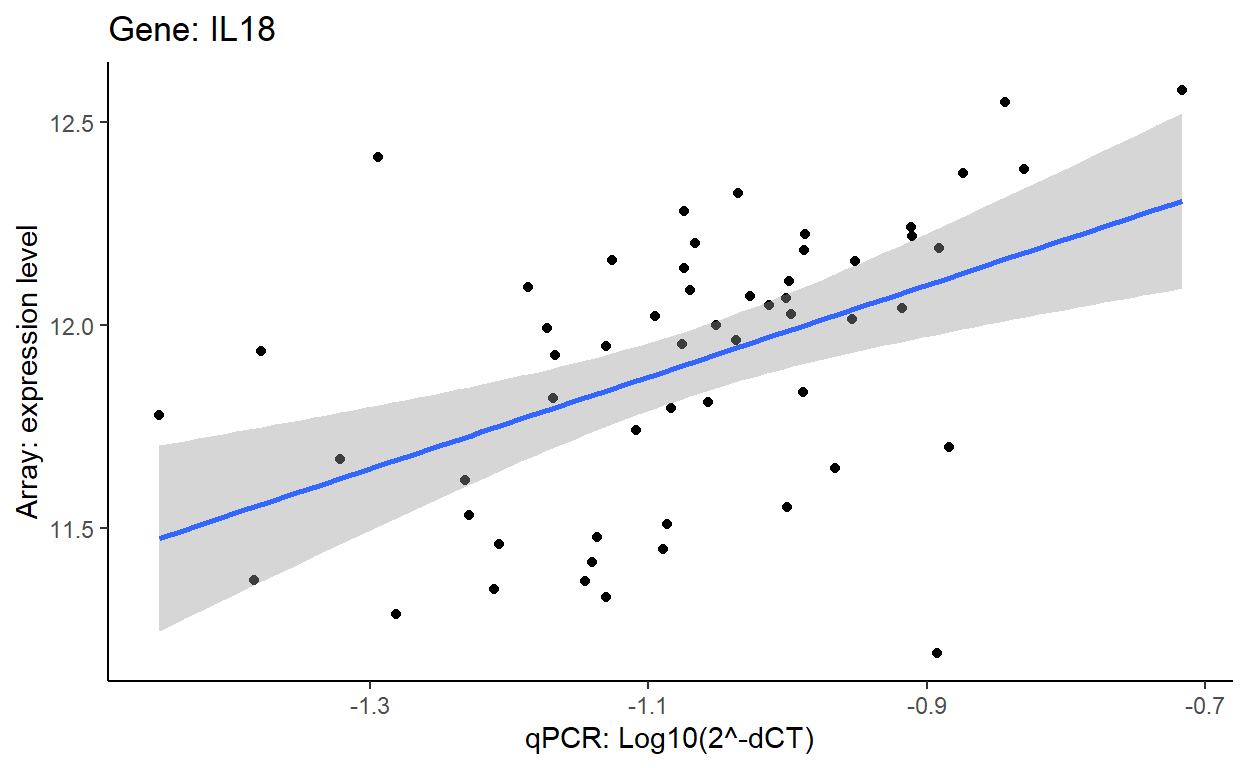 | Spearman's rank correlation rho  p-value = 3.295e-05  rho = 0.5317157 |

| 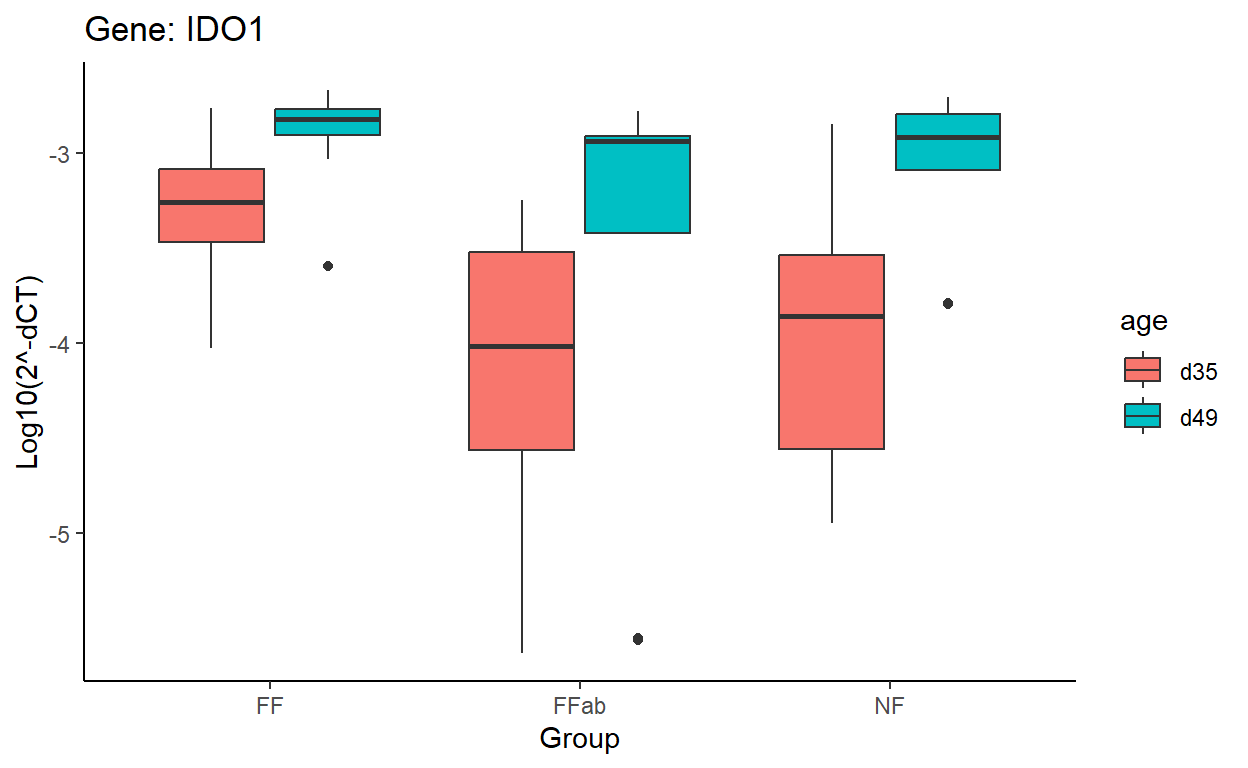 | 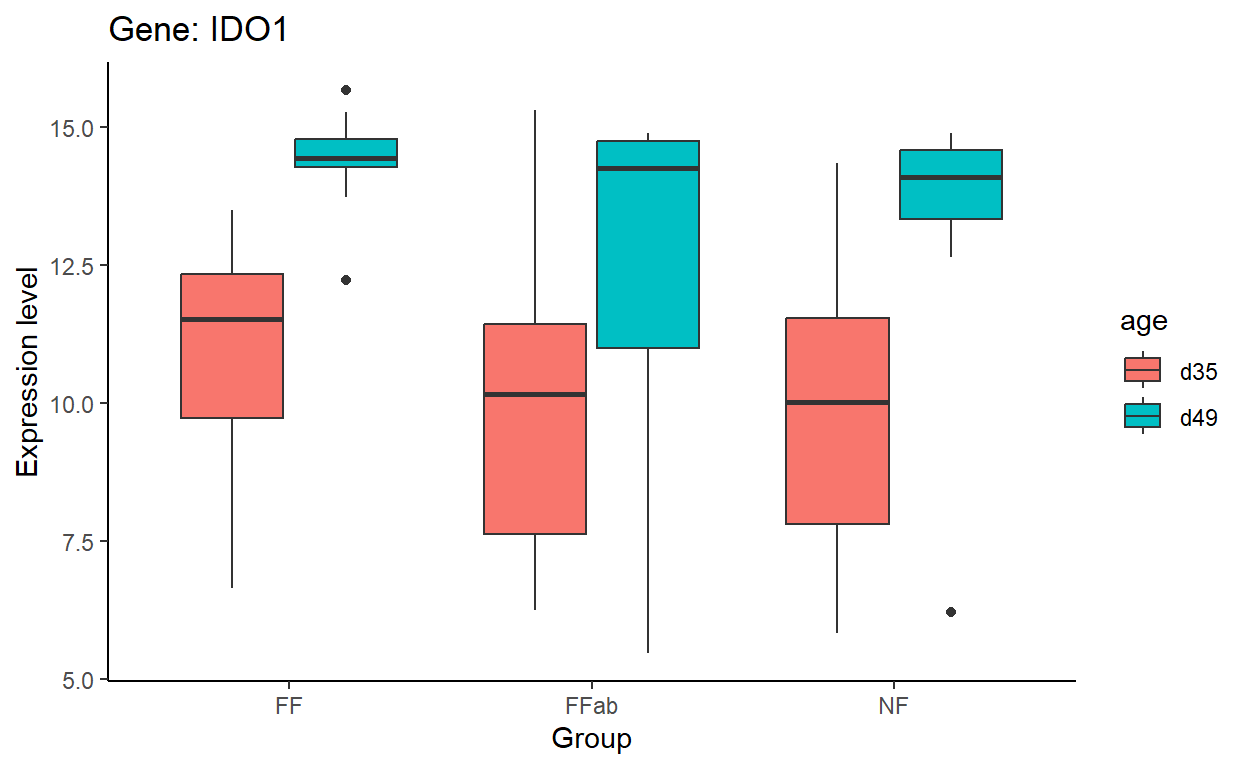 |
| --- | --- |
| 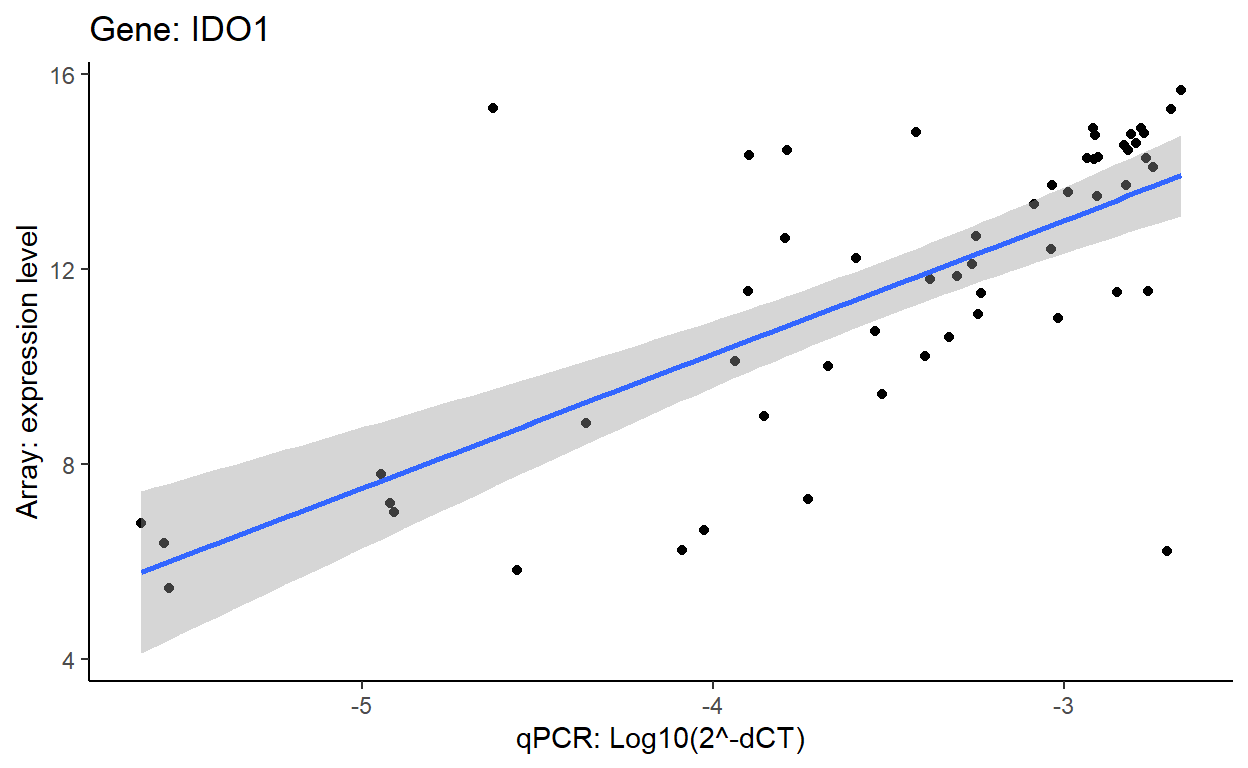 | Spearman's rank correlation rho  p-value = 1.139e-07  rho = 0.6392796 |

| 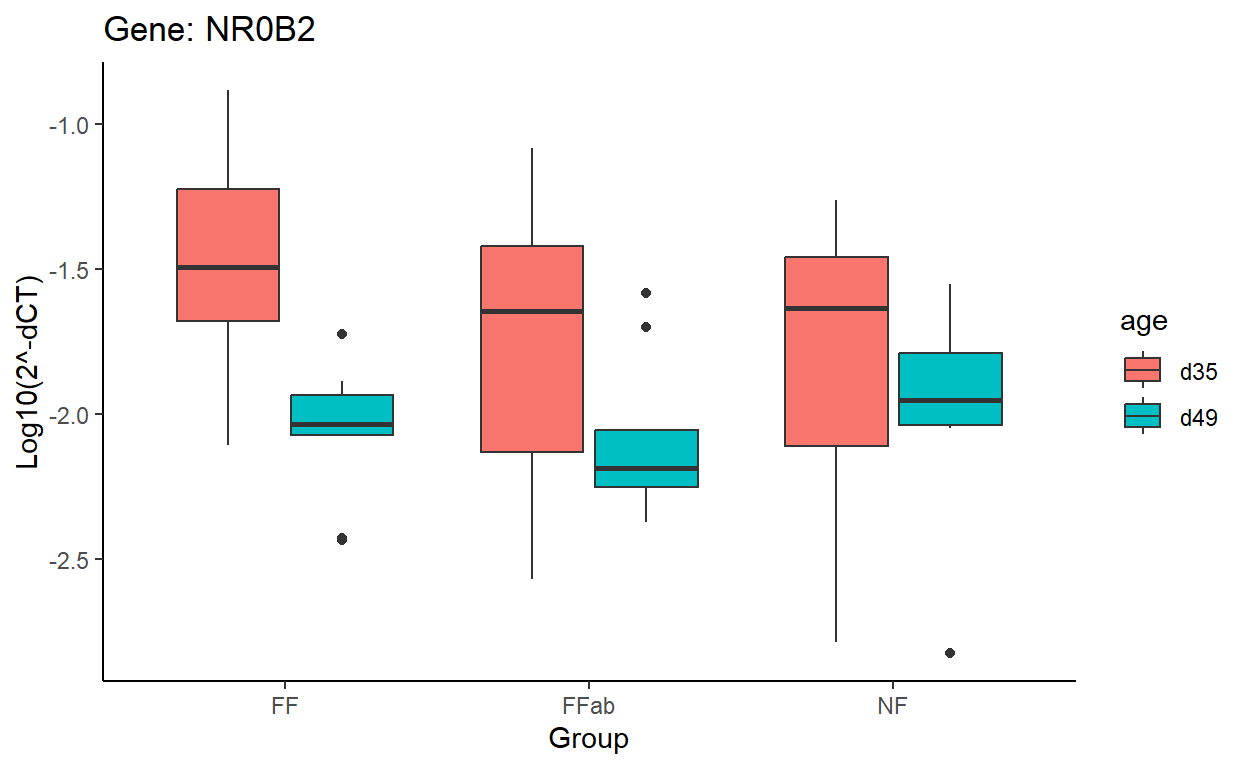 | 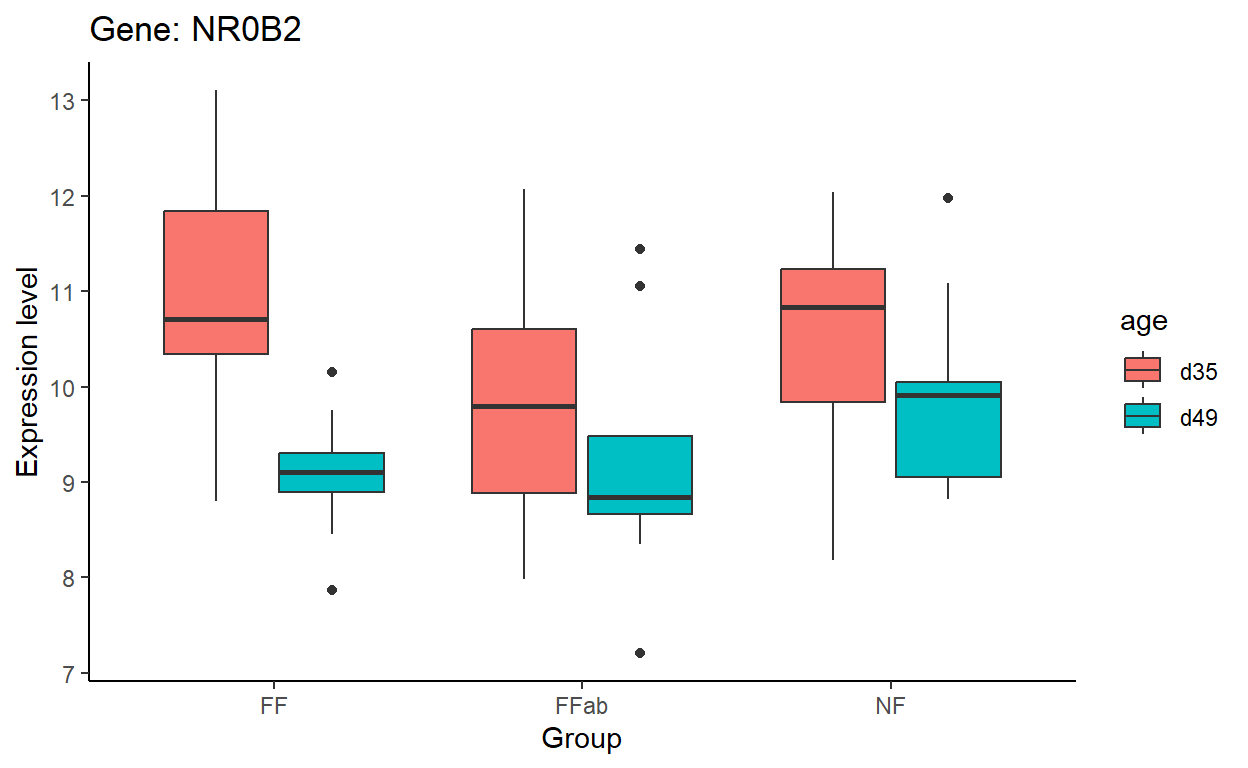 |
| --- | --- |
| 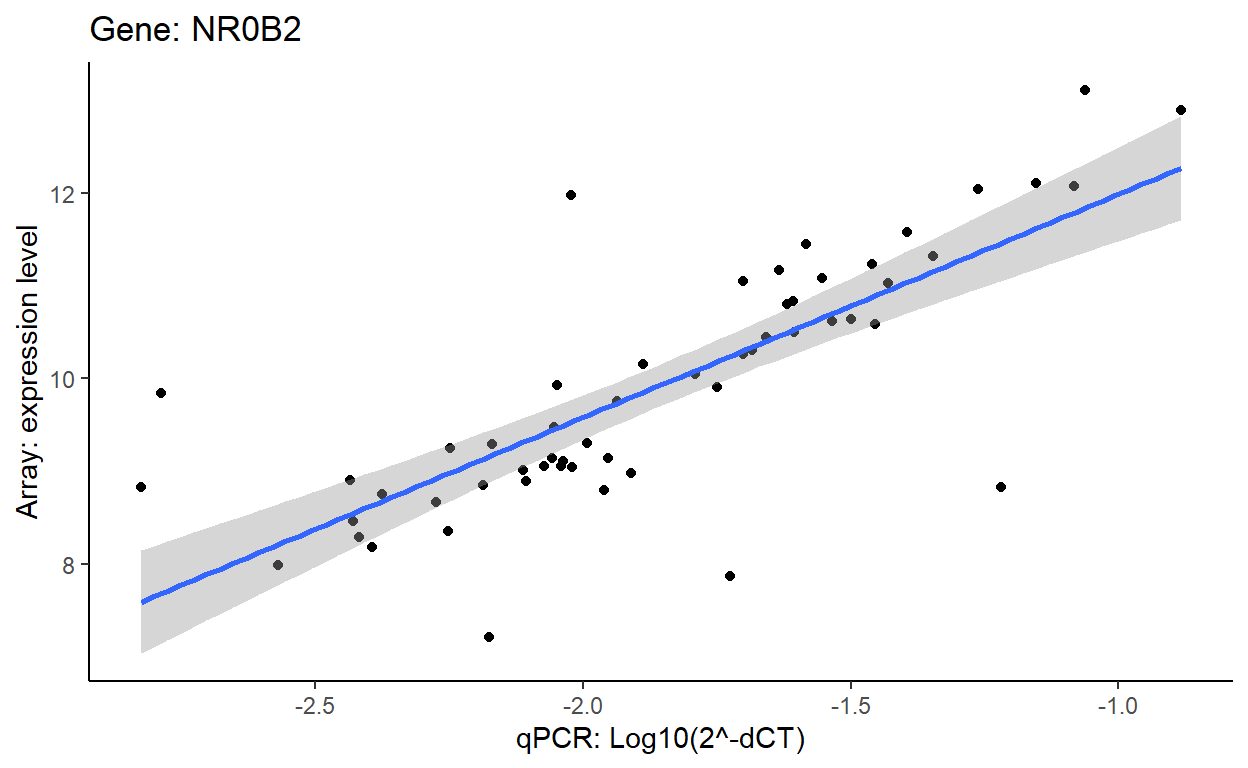 | Spearman's rank correlation rho  p-value = < 2.2e-16  rho = 0.7994532 |
