# Supplementary material for: Coprophagia in early life tunes expression of immune genes after weaning in rabbit ileum
Source: Sci Rep. 2024 Apr 17;14:8898. doi: 10.1038/s41598-024-59591-6 (PMC11024171; doi:10.1038/s41598-024-59591-6)
Supplement: Supplementary file 11 — Supplementary Information 10. [file 41598_2024_59591_MOESM11_ESM.docx]

**Additional file 1: Additional material for outlier illustrations, PCA of unique annotated expressed probe microarray data**. The file Additional_file1_outlier&PCA.docx is a world file, which contains a hierarchical clustering analysis of samples using 1-correlation as distance matrix and ward.D linkage, a nMDS plot of 1-corr matrix distance of the samples and a PCA of unique annotated expressed probe microarray data.

**Additional file 2: Workflow of annotation improvement of expressed genes of the microarray platform.** The file Additional_file2_pipeline.docx is a Word file, which describes the workflow improving annotation of the expressed genes using BetterBunny and manual BLAST on NCBI

**Additional file 3: List of the differentially expressed genes (|log2(fold change)|> 0.5 and p.adj value < 0.05) in rabbit ileum according to age (day 35 vs day 49).** The file Additional_file3_list_oligo_AGE.csv is a csv (comma-separated values) file which contains the differentially expressed genes in the ileum according to age (day 35 vs day 49) with a gene symbol approved by the HUGO Gene Nomenclature Committee or with and Ensembl gene identifier for rabbit genome (ENSOCUG) and associated probe name, probe sequence, Human Ensembl Gene identifier (ENSG), log2(fold-change) and P.adj.value.

**Additional file 4: List of the differentially expressed genes (|log2(fold change)|> 0.5 and p.adj value < 0.05) during the two weeks following weaning (day 35 vs day 49) in the ileum of rabbit that ingested feces from unrelated doe without antibiotic treatment (FF group : ingestion of Feces from non-medicated unrelated doe) in the nest during early life.** The file Additional_file4_list_oligo_d35vs49_FF.csv is a csv (comma-separated values) file which contains the differentially expressed genes in ileum with a gene symbol approved by the HUGO Gene Nomenclature Committee or with and Ensembl gene identifier for rabbit genome (ENSOCUG) and associated probe name, probe sequence, rabbit chromosome name location and Human Ensembl Gene identifier (ENSG)

**Additional file 5: List of the differentially expressed genes (|log2(fold change)|> 0.5 and p.adj value < 0.05) during the two weeks following weaning (day 35 vs day 49) in the ileum of rabbit that had no access to feces ingestion in early life in the nest (NF group : No Feces ingestion).** The file Additional_file5_list_oligo_d35vs49_NF.csv is a csv (comma-separated values) file which contains the differentially expressed genes in ileum with a gene symbol approved by the HUGO Gene Nomenclature Committee or with and Ensembl gene identifier for rabbit genome (ENSOCUG) and associated probe name, probe sequence, Human Ensembl Gene identifier (ENSG), log2(fold-change) and P.adj.value.

**Additional file 6: List of the differentially expressed genes (|log2(fold change)|> 0.5 and p.adj value < 0.05) during the two weeks following weaning (D35 vs D49) in the ileum of rabbit that ingested feces from doe with antibiotic treatment (FFab group : ingestion of Foreign Faeces from AntiBiotics treated unrelated doe) in the nest during early life.** The file Additional_file6_list_oligo d35vs49_FFab.csv is a csv (comma-separated values) file which contains the differentially expressed genes in ileum with a gene symbol approved by the HUGO Gene Nomenclature Committee or with and Ensembl gene identifier for rabbit genome (ENSOCUG) and associated probe name, probe sequence, Human Ensembl Gene identifier (ENSG), log2(fold-change) and P.adj.value.

**Additional file 7: Microbiota alpha, beta diversity and taxonomic composition in ileal content of rabbit at 35 and 49 days of age** in NF group, where ingestion of hard feces was prevented, in the FF and FFab groups where pups had access in the nest to feces excreted by foreign females receiving either no antibiotic or medicated with tiamulin and tetracycline. Additional_file7_ileal_microbiota.docx is a word file which provides graphical presentations of alpha, beta diversity and taxonomic composition of ileal content

**Additional file 8: List of GO terms enriched from the FF group annotated upregulated genes list (DE genes with Ensembl gene annotation for rabbit) using g:profiler**. The Additional_file8_list GO terms 35d-49d-FF up-regulated genes.csv file is a csv (comma-separated values) file which contains a list of GO terms enriched by g:Profiler in Biological Process (BP), Molecular Function (MP) and Cellular Content (CC) terms associated with their computed -log10(pvalue) and gene contribution indication.

**Additional file 9: IgA levels in ileal content of rabbit after weaning**. The file Additional_file9_ileal_IgA.docx is a word file which provides a graphical presentation of ileal IgA levels of young rabbits at day 35 and day 49 in the NF group, where ingestion of feces was prevented, in the FF and the FFab groups where pups had access in the nest to feces excreted by unrelated does receiving either no antibiotic or treated with tiamulin and tetracycline.

**Additional file 10:** **Primer sequences for qRT-PCR validation.** The file Additional_file10_qPCR_gene_expression_validation.docx is a Word file which contains gene names, description and primer sequences of the 14 genes used for qRT-PCR validation. Selected microarray or qRT-PCR gene expression graphs and correlations are also included.
